# Salt stress vs. salt shock - the case of sugar beet and its halophytic ancestor

**DOI:** 10.1186/s12870-019-1661-x

**Published:** 2019-02-06

**Authors:** Monika Skorupa, Marcin Gołębiewski, Katarzyna Kurnik, Janusz Niedojadło, Jacek Kęsy, Krzysztof Klamkowski, Katarzyna Wójcik, Waldemar Treder, Andrzej Tretyn, Jarosław Tyburski

**Affiliations:** 1 Centre for Modern Interdisciplinary Technologies, Nicolaus Copernicus University, Toruń, Poland; 2 Chair of Plant Physiology and Biotechnology, Faculty of Biology and Environment Protection, Nicolaus Copernicus University, Toruń, Poland; 3 Department of Cellular and Molecular Biology, Faculty of Biology and Environment Protection, Nicolaus Copernicus University, Toruń, Poland; 4 Research Institute of Horticulture, Skierniewice, Poland

**Keywords:** *Beta vulgaris*, RNA-Seq, Salt shock, Salt stress, Sea beet, Sugar beet, Transcriptome

## Abstract

**Background:**

Sugar beet is a highly salt-tolerant crop. However, its ability to withstand high salinity is reduced compared to sea beet, a wild ancestor of all beet crops. The aim of this study was to investigate transcriptional patterns associated with physiological, cytological and biochemical mechanisms involved in salt response in these closely related subspecies. Salt acclimation strategies were assessed in plants subjected to either gradually increasing salt levels (salt-stress) or in excised leaves, exposed instantly to salinity (salt-shock).

**Result:**

The majority of DEGs was down-regulated under stress, which may lead to certain aspects of metabolism being reduced in this treatment, as exemplified by lowered transpiration and photosynthesis. This effect was more pronounced in sugar beet. Additionally, sugar beet, but not sea beet, growth was restricted. Silencing of genes encoding numerous transcription factors and signaling proteins was observed, concomitantly with the up-regulation of lipid transfer protein-encoding genes and those coding for NRTs. Bark storage protein genes were up-regulated in sugar beet to the level observed in unstressed sea beet. Osmotic adjustment, manifested by increased water and proline content, occurred in salt-shocked leaves of both genotypes, due to the concerted activation of genes encoding aquaporins, ion channels and osmoprotectants synthesizing enzymes. bHLH137 was the only TF-encoding gene induced by salt in a dose-dependent manner irrespective of the mode of salt treatment. Moreover, the incidence of bHLH-binding motives in promoter regions of salinity-regulated genes was significantly greater than in non-regulated ones.

**Conclusions:**

Maintaining homeostasis under salt stress requires deeper transcriptomic changes in the sugar beet than in the sea beet. In both genotypes salt shock elicits greater transcriptomic changes than stress and it results in greater number of up-regulated genes compared to the latter. NRTs and bark storage protein may play a yet undefined role in salt stress-acclimation in beet. bHLH is a putative regulator of salt response in beet leaves and a promising candidate for further studies.

**Electronic supplementary material:**

The online version of this article (10.1186/s12870-019-1661-x) contains supplementary material, which is available to authorized users.

## Background

Sugar beet (*Beta vulgaris ssp. vulgaris*) is one of the most recently domesticated crops [1]. A wild ancestor of all beet crops is *Beta vulgaris ssp. maritima* (referred to as *B. maritima* or ‘sea beet’). This taxon occurs along the Mediterranean Sea coasts and along the Atlantic coasts of northern Europe [2, 3] and is well adapted to saline conditions [4, 5]. Consequently, sugar beet inherited certain salt-tolerance traits and is classified as a salt-tolerant glycophyte. However, *B. maritima* can tolerate salinity better than beet crop varieties. Cultivated beets display relatively high salt-sensitivity during germination and early seedling growth and under long-term exposure to high salinity [6, 7]. On the other hand, sea beet display considerable salinity tolerance during germination and early seedling development [8]. The analysis of relative growth rate parameters, performed by Rozema el al. [9], indicated that under two-week-long exposition to the salinity levels up to 300 mM NaCl, sugar beet shows slight, but significant reduction of salt tolerance with respect to *B. maritima*. The growth rate reduction of the sugar beet caused by high salinity was due to reduced leaf area, both at the whole plant level as well as that of the individual plant leaf. The resulting reduction in the capture of light is partly counteracted by the increase in leaf thickness and succulence during salt-acclimation in the sugar beet and yet, high succulence and increased leaf thickness essentially contribute to the salt tolerance in sea beet [9]. Taking into account the difference in salt tolerance between sugar beet and its wild relative, the possibility arises to identify the salt tolerance mechanisms which were lost during domestication. This goal can be achieved by comparing salt acclimation strategies in plants representing both genotypes. To date, such type of studies was performed mainly on tomato. They involved cultivars of *Solanum lycopersicum* and wild species such as *S. peruvianum* and *S. pimpinellifolium* [10, 11].

In this study, the sugar beet cv. Huzar was selected for comparisons with *B. maritima*. The ‘Huzar’ cultivar was extensively studied by our group in context of salt acclimation [12, 13]. During previous studies it was demonstrated that ‘Huzar’ differs from *B. maritima* in terms of salt response at both molecular and biochemical levels. Namely, we demonstrated that under long-term salt exposure the activation of certain responses may be delayed in leaves of *B. maritima* compared to cultivated beets, including cv. Huzar [12]. The differences in transcriptomic response to salinity between cv. Huzar and *B. maritima* manifested at the level of gene families, by different expression patterns of individual isoforms, as exemplified by PIP aquaporins [13]. Therefore, cv Huzar is a good choice when differences between a crop and its wild ancestor are sought for. At the same time, it is one of the most frequently cultivated varieties in Poland being kind of an ‘average’ variety in terms of agronomic traits.

As beet subspecies differ in salt tolerance, and due to the availability of full genome sequence that was recently published by Dohm et co-workers [14], the plant emerges as a model of choice for studies in this field. Sugar beet genome assembly released in May 2013 comprises 567 megabases, of which 85% were assigned to chromosomes. The number of protein-coding genes was predicted to be 27,421 [14]. Additionally, there are other sugar beet genome sequencing efforts available, including published genetic linkage maps, ESTs and BACs sequencing projects [15, 16]. Published reports on molecular mechanisms underlying salt response in sugar beet are scarce. The most comprehensive studies available, involving proteomic and transcriptomic approaches, were devoted to salt response of sugar beet M14, which is a salt-tolerant monosomic addition line, obtained from the intercross between *B. vulgaris* and *B. corolliflora* [17, 18].

Investigation of salt tolerance mechanisms requires a combination of high-throughput methods, such as RNAseq or proteomics, with morphological, physiological, cellular as well as biochemical analyses. Nevertheless, the method of salinity application has also to be carefully considered with respect to the interpretation of results. As suggested by Shavrukov [19], treatment with salinity may be executed in one of two forms, namely salt stress or shock. The essence of stress treatment is a gradual exposure to increasing salt levels. This approach is juxtaposed to shock, which consists in exposing plants instantly to a high level of salinity. The increase of salt concentration in nature usually occurs gradually, whereas salt shock is a relatively rare phenomenon [19]. Both stress and shock consist of osmotic and ionic component [20]. Only a few studies examined the effects of stress and shock in parallel experiments. Nevertheless, it was demonstrated that patterns of gene expression are different in response to these treatments [19], however, the mechanisms behind the reactions triggered by shock and stress, still remain an unexplored field.

The first goal of the study was to identify alterations in beet leaf transcriptome caused by acclimation to stress and response to shock. Second objective was to detect the salinity-related, genotype-specific traits in the patterns of gene expression in leaves of sea beet and sugar beet*.* The rationale behind this approach was to outline the overall strategy of acclimation to salinity in these closely related subspecies differing in salt tolerance. Another purpose was to identify the molecular mechanisms of acclimation to salinity, which might have been inherited from the halophytic progenitor of sugar beet, and discriminate them from the ones that were lost during domestication, or appeared in this process anew. Alterations in the leaf transcriptome due to the salt treatments were assessed by means of RNASeq. Concurrently, several physiological and biochemical parameters were measured, to provide the context for interpreting the salt treatment-induced changes in gene expression patterns.

## Results

The aim of this study was to compare transcriptional changes associated with physiological, cytological and biochemical mechanisms involved in salt response elicited by two modes of treatment in two beet subspecies differing in salt tolerance. To achieve this, we performed RNASeq analysis of leaves sampled from plants treated with gradually increasing NaCl levels (salt stress) or excised leaves challenged with acute salinity (salt shock).

### Effects of salinity on physiological, morphological, biochemical, cytological and chemical characteristics of plants

**Fig. 1:**
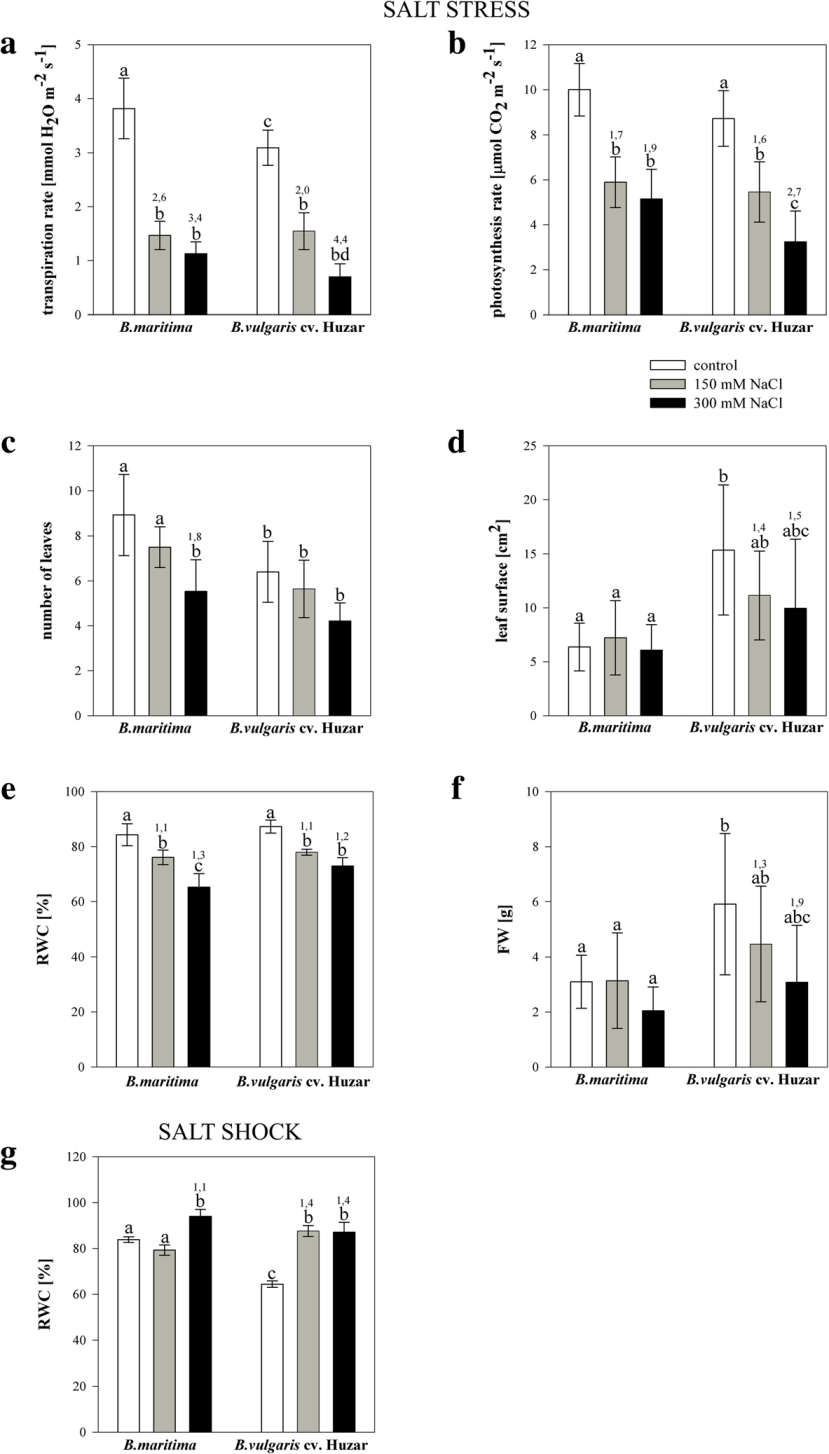
Morphological and physiological parameters determined for sea- (*B. maritima*) and sugar beet (*B. vulgaris cv.* ‘Huzar’) subjected to salt stress (**a-f**) or shock (**g**). Transpiration rate (**a**), photosynthesis rate (**b**), number of leaves (**c**), leaf surface (**d**), relative water content (RWC; e,g), fresh weight (FW; **f**). Numbers above the bars indicate fold changes relative to control. Different letters above the bars indicate significant differences at *p* < 0.01 (ANOVA followed by Tukey’s test)

A three-fold decrease in transpiration and in photosynthesis rate was detected in leaves of salt stress-acclimated plants, when compared to control plants (Fig. 1a,b). Moreover, water content was 1.2-fold decreased in leaves of stressed plants (Fig. 1e). Other parameters were not significantly affected by the treatment (Additional file 1: Figure S1 a,b). Salt-untreated sea beet excised leaves displayed 1.3-fold higher RWC than sugar beet ones (Fig. 1g). Leaf water content was increased 1.1 (*B. maritima*) and 1.4-fold (cv. *Huzar*) under high salinity, while under moderate shock it increased solely in sugar beet (1.4-fold). Photosynthetic rate was consistently below the detection limit (data not shown). Transpiration rate and CCI did not differ between the control and salt-shocked leaves (Additional file 1 Figure S1 c,d).

The number of leaves decreased 1.8-fold under strong salinity in *B. maritima* (Fig. 1c). Leaf area in sugar beet was 2.4-fold greater than in its wild ancestor, but decreased under strong stress, while in sea beet it remained constant (Fig. 1d).

**Fig. 2:**
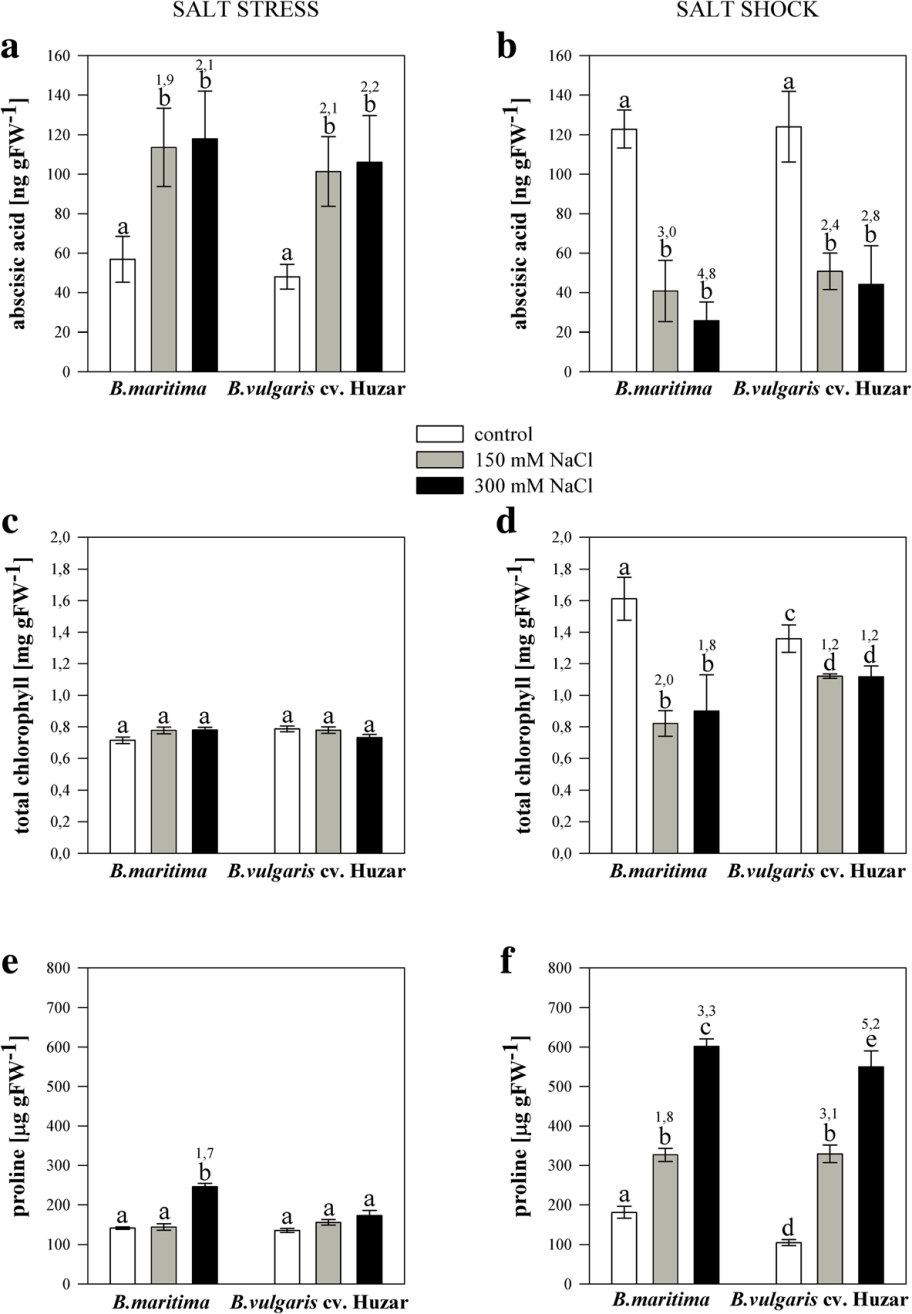
Biochemical parameters determined for sea- (*B. maritima*) and sugar beet (*B. vulgaris cv.* ‘Huzar’) subjected to salt stress (**a, c, e**) or shock (**b, d, f**). Abscisic acid (ABA; **a, b**), total chlorophyll (**c, d**) and proline (**e, f**). Numbers above the bars indicate fold changes relative to control. Different letters above bars indicate significant differences at p < 0.01 (ANOVA followed by Tukey’s test)

Acclimated plants displayed 2-fold increase of ABA concentration in leaves (Fig. 2a). The proline content did not change under moderate stress in both genotypes. Under strong salinity proline content increased 1.7-fold in the halophytic beet but in sugar beet stayed at the control level (Fig. 2e). In excised leaves ABA level was 4-fold higher than in intact control plants (Fig. 2b), but salt treatment abolished this effect (Fig. 2b). Both genotypes displayed increased proline content under salinity, and the osmoprotectant concentration was elevated by salt in a dose-proportional manner (2.5 and 4-fold, respectively; Fig. 2f). Conversely, the chlorophyll content decreased in salt-shocked leaves of both sea- and sugar beet (2 and 1.2-fold, respectively; Fig. 2d).

**Fig. 3:**
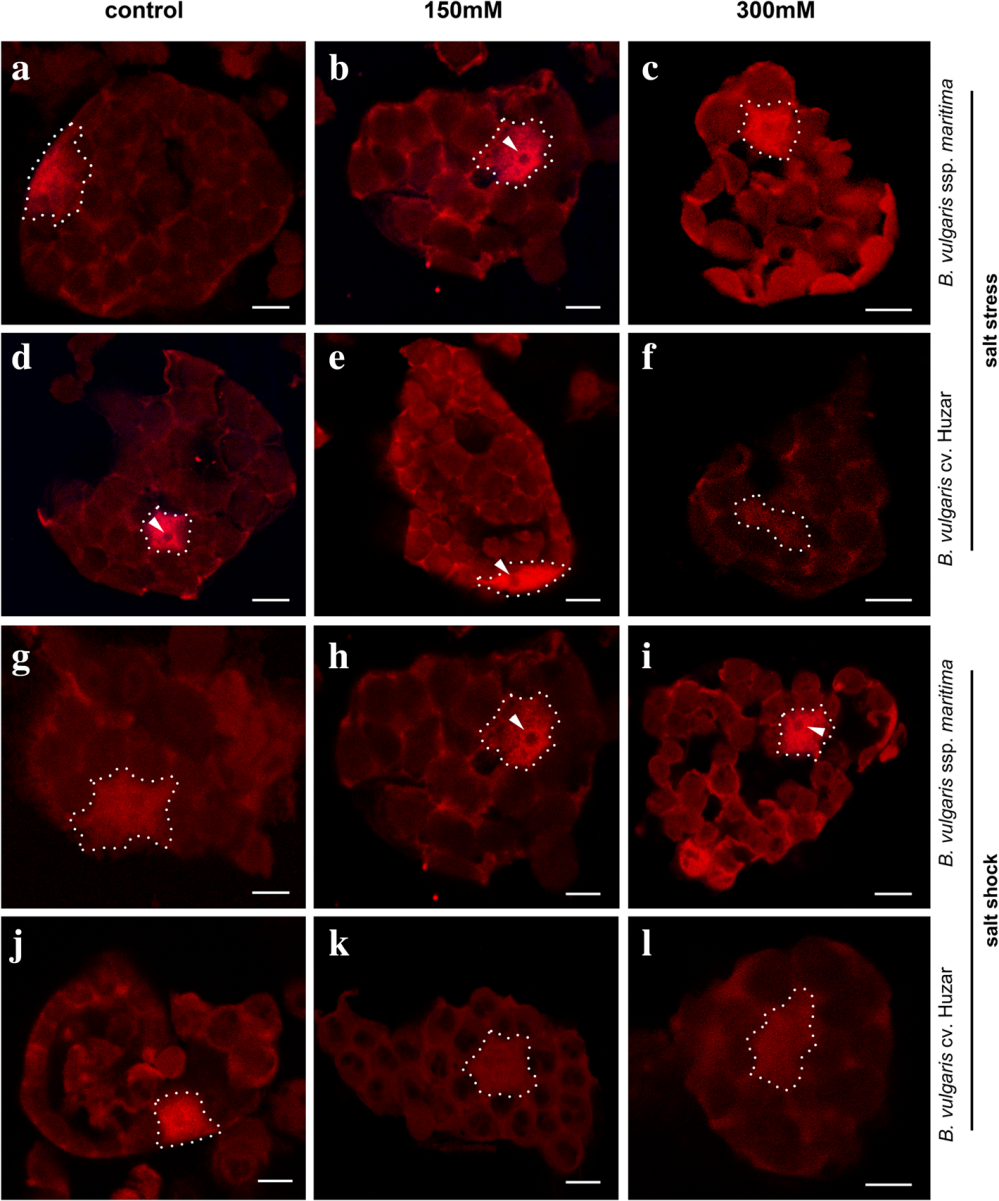
Localization of polyA RNA (red fluorescence) determined for mesophyll cells of sea- (*B. maritima* – **a-c** and **g-i**) and sugar beet (*B. vulgaris cv.* ‘Huzar’ – **d-f** and **j-l**) subjected to salt stress (**a-f**) or shock (**g-l**). Control (**a, d, g, j**), 150 mM NaCl treatment (**b, e, h, k**), and 300 mM NaCl treatment (**c, f, i, l**). Nuclei are surrounded by dotted line. Arrow head – nucleoli; Scale bar: 5 μm

**Fig. 4:**
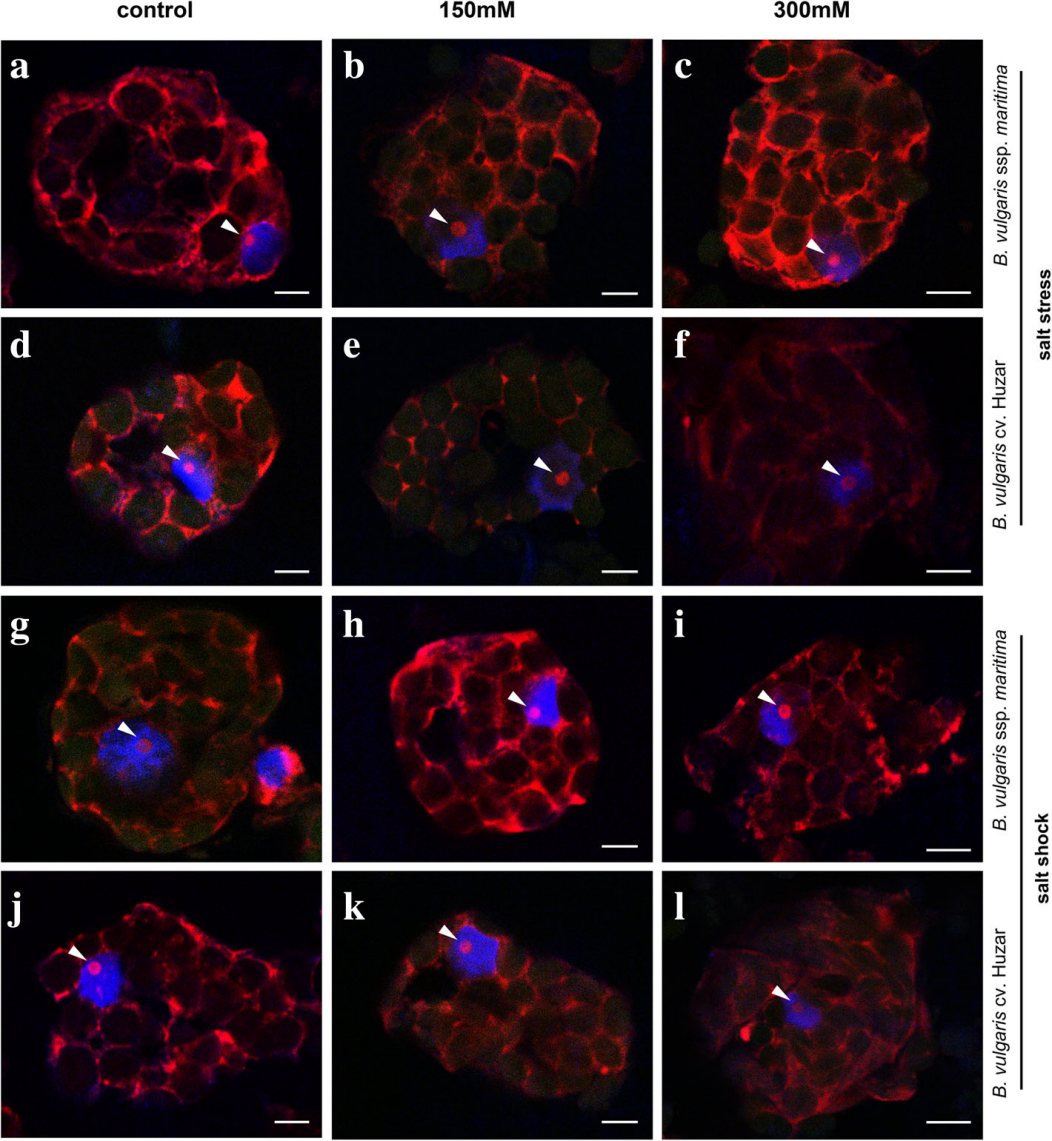
Localization of rRNA (red fluorescence) determined for mesophyll cells of sea- (*B. maritima* – **a-c** and **g-i**) and sugar beet (*B. vulgaris cv.* ‘Huzar’ – **d-f** and **j-l**) subjected to salt stress (**a-f**) or shock (**g-l**). Control **(a, d, g, j**), 150 mM NaCl treatment (**b, e, h, k**), and 300 mM NaCl treatment (**c, f, i, l**). Merged with DAPI staining (blue) to indicate nuclei. Arrow head – nucleoli. Scale bar: 5 μm

**Fig. 5:**
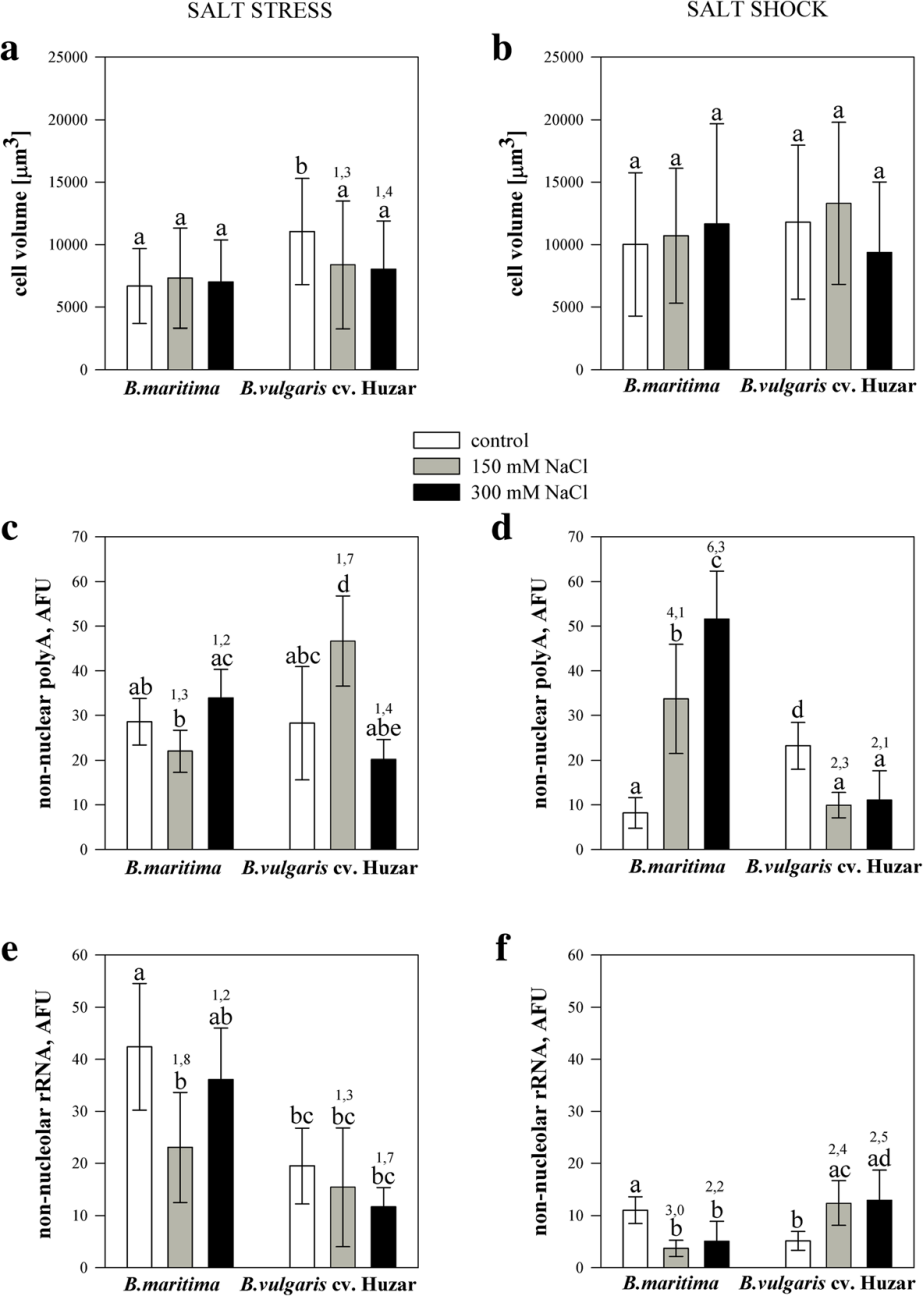
Mesophyll cell parameters for sea- (*B. maritima*) and sugar beet (*B. vulgaris cv*. ‘Huzar’) subjected to salt stress (**a, c, e**) or shock (**b, d, f**). Cell volume (**a, b**), non-nuclear polyA RNA (**c, d**), non-nucleolar rRNA (**e, f**). AFU – arbitrary fluorescence units. Fluorescence from individual cells was divided by cell volume. Numbers above the bars indicate fold changes relative to control. Different letters above bars indicate significant differences at *p* < 0.01 (ANOVA followed by Tukey’s test)

PolyA RNA level was used as an approximation of transcription rate, while non-nucleolar rRNA served as a proxy for potential translational capacity. Polyadenylated RNA was detected in the nucleus and cytoplasm. The homogeneous signal was detected in the nucleoplasm, except the nucleolus. In cytoplasm, the fluorescence was weaker and present mostly in the spaces between chloroplasts (Fig. 3). In control cells the signal representing 25S rRNA was identified in the cytoplasm. The pattern of distribution of rRNA in *B. maritima* mesophyll cells did not change due to salt stress nor shock. However, in salt-stressed sugar beet rRNA did not fill the whole cytoplasm and sometimes occurred in cytoplasmic granules (Fig. 4). Sugar beet cell volume was 1.4-fold greater than in *B. maritima* in control plants, and this difference disappeared under stress, due to the decrease of crop’s cell size (Fig. 5a). The quantity of polyadenylated RNA in the cytoplasm of leaf cells of *B. maritima* slightly decreased under moderate stress, whereas in sugar beet the parameter was 1.7-fold increased by moderate stress and decreased by the strong one below the control level (Fig. 3 and 5c). The rRNA content in both genotypes was lowered by moderate stress (1.8-fold in *B. maritima* and 1,3-fold in cv. *Huzar*). *B. maritima* cells under strong stress displayed rRNA level similar to control, while in sugar beet it remained low (Fig. 4 and 5e). No significant differences in cell volume under shock were observed when compared to control, however an increasing trend was observed in *B. maritima.* In the sugar beet this tendency was visible under moderate shock only (Fig. 5b). The level of polyadenylated RNA increased (4 and 6-fold under moderate and strong shock, respectively) in *B. maritima* and decreased 2-fold in the sugar beet (Fig. 4 and 5d). Oppositely, rRNA content was increased (2.5-fold) in shocked the sugar beet and decreased (2.6-fold) in the sea beet (Fig. 4 and 5f).

Sodium and chlorine levels were increased (~ 2 and 10-fold, respectively) in leaves coming from plants challenged with salt stress, regardless of genotype and stress intensity. Both genotypes displayed decreased level of potassium under stress, however in sugar beet the decrease was salt concentration-dependent (1.4 and 1.6-fold, respectively) whereas in sea beet the K content was lowered (1.3-fold) to the same level regardless of the stress intensity. Other elements were not influenced by salinity irrespective of genotype and salt concentration (Additional file 1: Figure S2). Situation was analogical under salt shock treatment (Additional file 2: Figure S3) Physiochemical parameters of growth substrate solution are described in Additional file 2 and shown in Additional file 1: Figure S4.

### Overview of changes in beet leaf transcriptome under salt stress or shock

**Fig. 6:**
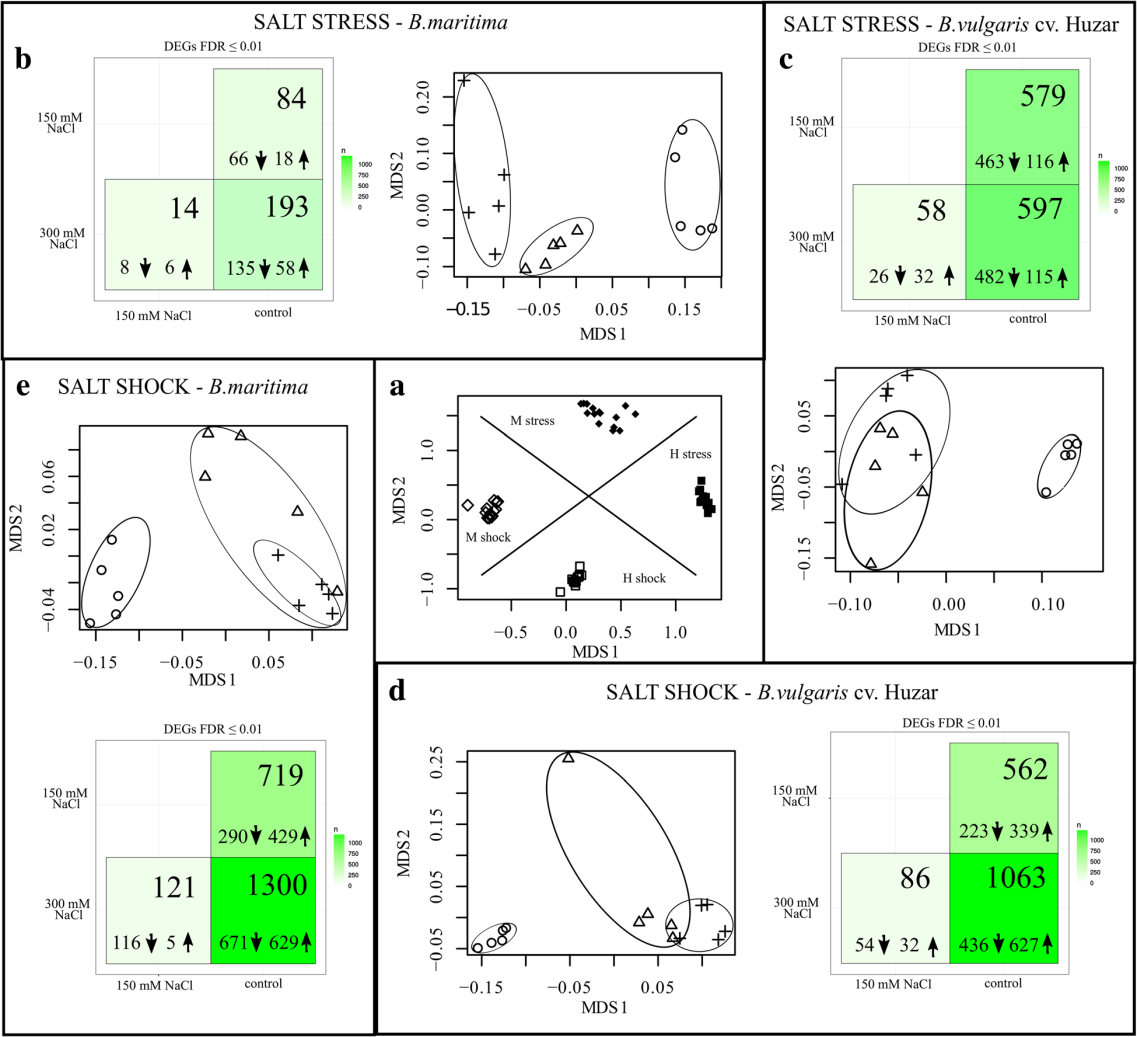
NMDS/PERMANOVA analysis of Morisita-Horn distance matrix generated from RNAseq data and heatmaps showing numbers of DEGs. Beet leaves transcriptomes differed significantly according to the mode of salt treatment and genotype (*p* < 0.01). M – *B. maritima*, H – *B. vulgaris ssp. vulgaris cv* ‘Huzar’ (**a**). Heatmaps in panels **b-e** show numbers of total numbers of DEGs (FDR < 0.01, upper part of each square) and numbers of up- (upward arrows) as well as down-regulated (downward arrow) ones. Ordination plots in b-e panels show separation of samples according to the concentration of salt in a given treatment. Circles denote controls (no salt), triangles – 150 mM NaCl, crosses – 300 mM NaCl. In each case controls were significantly different from salt-treated samples, while there were no significant differences between the latter. Note difference in scales between panel a and the remaining ones

Samples (sets of DEGs) clustered tightly according to the mode of salt treatment and genotype (Fig. 6a). Differences between samples groups turned out to be significant according to PERMANOVA (*p* < 0.01). Irrespectively of salt concentration, DEGs sets representing salt-stressed or shocked plants were highly dissimilar from the controls. On the other hand, the differences were much smaller and insignificant (*p* = 0.07) when the effects of two intensities of salt stress were compared (Fig. 6b-e).

The number of DEGs identified in plants representing particular experimental variant was strongly affected by plant genotype, mode of salt treatment and salt concentration. More DEGs were identified in salt stress-acclimated sugar beet as compared to *B. maritima,* whereas under salt shock, roughly similar number of transcripts was differentially regulated in both genotypes. The response to shock brought about larger scale of transcriptional rearrangements than acclimation to salt stress, and strong salt stress or shock resulted in higher number of DEGs compared to moderate salinization. The leaves of salt-stressed plants were characterized by markedly higher numbers of down-regulated, than up-regulated transcripts. On the other hand, a minor difference between the number of down- and up-regulated DEGs was observed in salt-shocked leaves (Fig. 6b-e). Sequencing statistics is shown in Additional file 1: Table S1. Expression levels (in FPKM) for all transcripts identified in the study are shown in Additional file 3.

### Common and genome-specific traits of the transcriptomic response to salt stress and salt shock in beet leaves

**Fig. 7:**
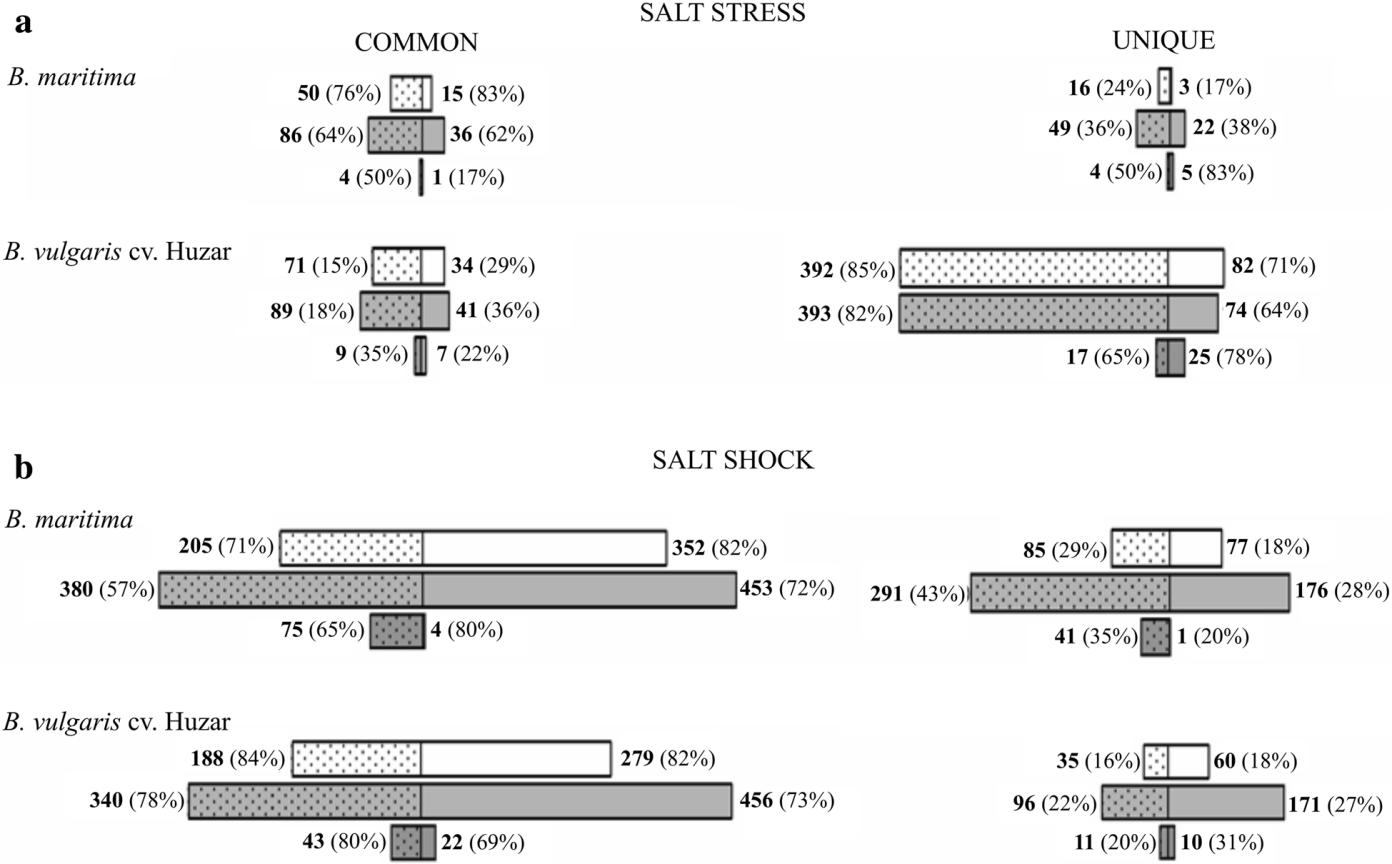
Distribution of DEGs among ‘COMMON’ and ‘UNIQUE’ categories, data for stress (**a**) and shock (**b**). ‘COMMON’ – genes that were differentially expressed both in sea- and sugar beet, ‘UNIQUE’ – genes that were differentially expressed in one of the genotypes only. White bars - controls vs.150 mM NaCl treatment comparison, grey bars - controls vs. 300 mM NaCl, dark grey bars - 150 mM vs. 300 mM NaCl. Dotted parts of bars represent down-regulated genes, while clear parts denote up-regulated ones. Bold-faced numbers denote up- or down-regulated genes within a given category and comparison. Percentage values given are relative to total numbers of up- or down-regulated genes in a given comparison

Among differentially expressed transcripts three groups were distinguished: i) ‘common’ – transcripts whose expression changed significantly in both genotypes, hallmarking traits possibly inherited by the sugar beet from its wild ancestor (Additional file 4), ii) ‘sugar beet-specific’ – transcripts whose expression changed in sugar beet only, representing traits acquired during domestication (Additional file 5) and iii) *B. maritima*-specific – those whose expression changed in *B. maritima* only, representing traits lost during domestication (Additional file 5). This step of analysis involved the identification of transcripts (*with the same annotation*) differentially expressed either in both genotypes (“common” DEGs) or specifically in one of the two genotypes (“unique” DEGs). The “common” and “unique” DEGs were identified separately for plants subjected to salt stress and salt shock (Additional files 4, 5, Fig. 7).

The majority (66%) of salt stress-affected DEGs in *B. maritima* were classified as “common” DEGs. They were more common among the up-regulated transcripts, than among the down-regulated ones under moderate stress, while under strong stress the values were almost identical (Fig. 7). Salt stress acclimation in sugar beet resulted in more DEGs than in *B. maritima*. Consequently, the sugar beet DEGs assemblage was largely composed of “unique” ones. Nevertheless, the observed tendency was similar to this found in *B. maritima*. Under salt-shock, irrespective of genotype and salt concentration, the majority of DEGs was common. Their percentage was consistently higher for the up-regulated transcripts (Fig. 7).

### Gene ontology enrichment analysis

GO terms that passed the significance threshold of *P* ≤ 0.001 were operationally classified into ten process-related categories encompassing terms coming from all three supercategories (Tables 1 and 2). We omitted general terms, such as ‘cation binding’ or ‘cellular metabolism’.

**Table 1:** GO categories of DEGs identified in leaves of beet plants under salt stress

	150 mM NaCl		300 mM NaCl	
	*B. maritima*	*B. vulgaris* cv. ‘Huzar’	*B. maritima*	*B. vulgaris* cv. ‘Huzar’
Biological Process	proline catabolism to glutamate (1,7), polysaccharide catabolism (3,7), triterpenoid biosynthesis (2),	cellular glucan metabolism (3,7), response to chitin (5), protein ubiquitination (4), trehalose biosynthesis (3,7), cellular iron ion homeostasis, defense response (5), camalexin biosynthesis (5), protein phosphorylation (4,6), L-serine biosynthesis (1), response to biotic stimulus (5), circadian rythm	pathogenesis (5), proline catabolism to glutamate (1,7), methionine catabolism (1), protein polymerisation (4)	protein ubiquitination (4), cellular glucan metabolism (3,7), trehalose biosynthesis (3,7), carbohydrate metabolism (3,7), response to wounding (5), inositol trisphosphate metabolism (3,6), response to chitin (5), camalexin biosynthesis (5), protein phosphorylation (4,6)
Molecular Function	β-amylase (3,7), malate synthase, 1-pyrroline-5-carboxylate dehydrogenase (1,7), hydrolase, hydroxymethyl-glutaryl-CoA reductase, protochlorophyllide reductase (10), **galactose transmembrane transporter** (9), endopeptidase inhibitor (4)	protein tyrosine kinase (6), xyloglucan:xyloglucosyl transferase (3,8), transcription factor (6), Ca^2+^ binding (6), protein serine/threonine kinase (6), ubiquitin protein transferase (4), **galactose transmembrane transporter** (9), UDP-glucuronate 4 epimerase (3,8), Fe^2+^ binding, aspartic type endopeptidase (4), calmodulin binding (6)	catechol oxidase (8), β-amylase (3,7), 1-pyrroline-5-carboxylate dehydrogenase (1,7), glutamate 5-semialdehyde dehydrogenase (1), methionin γ-lyase (1)	protein tyrosine kinase (4,6), xyloglucan:xyloglucosyl transferase (3,8), protein serine/threonine kinase (4,6), ubiquitin protein transferase (4), Ca^2+^ binding (6)
Cellular Compartment	–	ubiquitin ligase complex (4), **plant-type cell wall** (8), integral component of membrane (9)	**plant-type cell wall** (8)	**plant-type cell wall** (8), ubiquitin ligase complex (4), proteinaceous extracellular matrix (8), apoplast (8), integral component of membrane (9)

1 – aminoacids catabolism, 2 – secondary metabolism, 3- carbohydrates metabolism, 4 – protein processing, 5 – pathogen response, 6 – transcription regulation and signalling, 7 – osmoprotectant metabolism, 8 – cell wall plasticity, 9 – transmembrane transport, 10 – photosynthesis

GO terms were required to pass significance threshold of *P* ≤ 0.001

The GO categories, common for *B. maritima* and *B. vulgaris* cv. 'Huzar' are marked in bold

**Table 2:** GO categories of DEGs identified in beet leaves under salt shock

	150 mM NaCl		300 mM NaCl	
	*B. maritima*	*B. vulgaris* cv. ‘Huzar’	*B. maritima*	*B. vulgaris* cv. ‘Huzar’
Biological Process	**Carbohydrate metabolism** (3,7), trehalose biosynthesis (3,7), cellulose biosynthesis (3,8), water transport (9), extracellular polysaccharide biosynthesis (3,8), **cellular glucan metabolism** (3,7), pathogenesis (5), \polysaccharide catabolism (3,7)	Response to chitin (5), protein ubiquitination (4), **cellular glucan metabolism** (3,7), defense response (5), camalexin biosynthesis (5), response to biotic stimulus (5), protein phosphorylation (4,6), L-serine biosynthesis (1)	**Carbohydrate metabolism** (3,7), photosynthesis (10), photosynthesis – light harvesting (10), ATP hydrolysis-coupled proton transport (9), cellulose biosynthesis (3,8), ATP synthesis-coupled proton transport (9,10), trehalose biosynthesis (3,7), chlorophyll biosynthesis (10), response to karrikin, extracellular polysaccharide biosynthesis (3,8), response to oxidative stress, water transport (9), phenylpropanoid metabolism (2), **nucleoside metabolism**, thiamine biosynthesis	**Carbohydrate metabolism** (3,7), inositol trisphosphate metabolism (3,6), camalexin biosynthesis (5), protein ubiquitination (4), cellular glucan metabolism (3,7), response to chitin (5), **nucleoside metabolism**
Molecular Function	Cellulose synthase (UDP-forming) (3,8), β-galactosidase (3), dTDP-4-dehydrorhamnose reductase (3,8), **xyloglucan:xyloglucosyl transferase** (3,8), inosine nucleosidase, uridine nucleosidase, protochlorophyllide reductase (10), acid phosphatase, **UDP-glucuronate 4 epimerase** (3,8), α-L-arabinofuranosidase (3,8), transporter (9)	Protein tyrosine kinase (4,6), **xyloglucan:xyloglucosyl transferase** (3,8), Ca^2+^-binding (6), protein serine/threonine kinase (4,6), transcription factor (6), Fe^2+^-binding, **UDP-glucuronate 4 epimerase** (3,8), ubiquitin protein transferase (4), aspartic-type endopeptidase (4), calmodulin binding (6)	Cellulose synthase (UDP-forming) (3,8), dTDP-4-dehydrorhamnose reductase (3,8), β-galactosidase (3), transmembrane transporter (9), protochlorophyllide reductase (10), chorismate mutase (1), symporter (9), aldehyde dehydrogenase, acid phosphatase, proton transporting ATPase (9), polyU RNA-binding, secondary active sulfate, NAD-binding, extracellular glutamate-gated ion channel (9), ionotropic glutamate receptor (6), proton-transporting ATP synthase (9,10)	Protein tyrosine kinase (4,6), **xyloglucan:xyloglucosyl transferase** (3,8), protein serine/threonine kinase (4,6), Ca^2+^-binding (6), transcription factor (6), **UDP-glucuronate 4 epimerase** (3,8), triglyceride lipase, β-galactosidase (3), ubiquitin protein transferase (4)
Cellular Compartment	**Plant-type cell wall** (8), extracellular region (8), apoplast (8), **proteinaceous extracellular matrix** (8), integral component of membrane (9), cell wall (8), chloroplast thylakoid membrane (10)	ubiquitin ligase complex (4), **plant-type cell wall** (8), extracellular matrix (8), Golgi-cisterna membrane	chloroplast thylakoid membrane (10), plastoglobule, **plant-type cell wall** (8), plasma membrane (9), photosystem I reaction center (10), apoplast (8), integral component of membrane (9), chloroplast envelope (10), proton transporting two-sector ATPase complex (9,10), **proteinaceous extracellular matrix** (8), COPI vessicle coat, photosystem II antenna complex (10), plastid large ribosomal subunit (10), chloroplast photosystem I (10), chloroplast stroma (10)	**plant-type cell wall** (8), **proteinaceous extracellular matrix** (8), ubiquitin ligase complex (4), apoplast (8), extracellular matrix (8), Golgi cisterna membrane

1 – aminoacids metabolism, 2 – secondary metabolism, 3- carbohydrates metabolism, 4 – protein processing, 5 – pathogen response, 6 – transcription regulation and signalling, 7 – osmoprotectant metabolism, 8 – cell wall plasticity, 9 – transmembrane transport, 10 – photosynthesis

GO terms were required to pass significance threshold of P ≤ 0.001

The GO categories, common for *B. maritima* and *B. vulgaris* cv. 'Huzar' are marked in bold

Salt stress. Terms belonging to ‘osmoprotectant metabolism’ and ‘transmembrane transport’ categories were common for sea- and sugar beet. ‘Amino acid metabolism’, ‘secondary metabolism’ and ‘photosynthesis’ were characteristic for *B. maritima*, while ‘carbohydrates metabolism’, ‘protein processing’, ‘pathogen response’, ‘transcription regulation and signaling’ as well as ‘cell wall plasticity’ were specific for sugar beet (Table 1).

#### Salt-shock

Just as more DEGs were identified in salt shock than in stress, more categories were significant in GO enrichment. Generally, more categories were found significant in *B. maritima* than in sugar beet. Terms from ‘amino acid metabolism’, ‘carbohydrates metabolism’, ‘osmoprotectant metabolism’ and ‘cell wall plasticity’ were common for sea- and sugar beet. ‘Secondary metabolism’, transmembrane transport’ and ‘photosynthesis’ distinguished sea beet, while ‘protein processing’, ‘pathogen response’ as well as ‘transcription regulation and signaling’ were characteristic for sugar beet (Table 2).

Hereafter, when we refer to a protein or gene name, we mean a transcript encoded by this gene or coding for the given protein.

### Differences in transcriptome composition between non-stressed sea- and sugar beet

In the absence of stress, the genotypes displayed small, albeit visible, differences in the transcriptome composition. Seventy-seven DEGs were up-regulated in *B. maritima* with respect to sugar beet. Sugar beet leaves were marked by increased levels of 71 transcripts. In both genotypes, the majority of annotated transcripts belonged to the following categories: cell wall building/plasticity, pathogen response/biotic stress, signaling and growth regulation, however their proportions differed. The ensemble of transcripts over-expressed in *B. maritima* was markedly enriched in DEGs encoding proteins involved in pathogen response as well as cell wall building (cellulose synthase and proline-rich protein 4) and were characterized by higher expression of transcripts encoding bark-storage protein. Very high expression level of a transcript encoding DDT domain-containing protein PTM, functioning in signal transduction from chloroplast to nucleus [21], was observed in both genotypes, but higher level was seen in sea beet. On the other hand, cell wall loosening enzymes (xyloglucan hydrolases) and metallothiol transferase were hallmarking sugar beet. In non-stressed sugar beet, the bark storage protein-encoding transcripts were weakly expressed. However, they were strongly up-regulated under salt stress in sugar beet leaves (Additional file 1: Table S2).

### Analysis of selected transcripts differentially expressed under salt stress or shock

To further reduce complexity of our data we selected representative differentially expressed transcripts belonging to i) the most significant GO categories representing three groups discriminated based on genotype specificity that was mentioned above, ii) those not belonging to significant GO categories but highly responsive to salinity and iii) those representing novel mechanisms involved in salinity response. The information on the transcripts was placed in (Additional file 1: Table S3).

#### Transcription regulation and signaling

Among the transcripts common to sugar- and sea beet, bHLH137 was the only TF up-regulated by salt and the same time the one most affected (in terms of the fold change) by this stressor. The response was dose-dependent and more pronounced under shock than under stress. TFs from WRKY, NAC, MYB or SCARECROW families were specifically down-regulated in sugar beet under stress (Additional file 1: Table S3). THESEUS-1 and FERONIA receptor kinases involved in regulation of cell wall synthesis were specifically silenced in sugar beet under stress, while their expression increased in both genotypes under shock. Many other kinases and phosphatases were down-regulated in sugar beet under stress, with several exceptions, such as D6P kinase or phosphatase 2C 24. Transcripts encoding proteins involved in calcium-mediated signaling, such as those of the calcineurin B family, were up-regulated in sea beet under shock.

#### Osmoprotectants metabolism

The expression patterns of important genes involved in osmoprotectant metabolism differed between genotypes. δ-1-pyrroline-5-carboxylate synthase-(P5CS) and betaine aldehyde dehydrogenase (BADH) involved in synthesis of osmoprotectants were up-regulated in sea beet plants acclimated to strong salt stress and in both genotypes under shock. Expression of δ-1-pyrroline-5-carboxylate dehydrogenase (P5CDH), engaged in proline degradation, was increased under stress. Furthermore, sucrose synthase was up-regulated under salt shock.

#### Transmembrane transport

Salt stress treatments generally resulted in decreased expression of aquaporins and ion-channels, while shock brought about their up-regulation. Certain exceptions were found, such as probable voltage-gated potassium channel up-regulated under stress in the sea beet. Interestingly, several genes encoding ion channel proteins (e.g. HKT1 or KAT1) were down-regulated specifically in sugar beet under stress. The response to salt stress was marked by increased expression of genes encoding lipid transfer proteins. Contrastingly, these transcripts were down-regulated under salt shock. Sugar transporters were either down-regulated (e.g. sugar transporter 14, SWEET14 in both genotypes or sugar transporter 1, SWEET2 and SWEET12 in sugar beet) or not affected (ERD6, SUC3) by salt stress and up-regulated under salt shock, with certain exceptions.

#### Cell wall plasticity

Genes coding for proteins engaged in cell wall flexibility (expansins, arabinogalactan proteins, xyloglucan hydrolases) were generally silenced under stress and up-regulated under shock, however the silencing was more pronounced in sugar beet. Cell wall synthesis-involved genes were either down-regulated under stress and up regulated under shock (cellulose synthase) or displayed the opposite behavior (cynamoyl reductase). From among genes regulating cell wall functions, members of the EXORDIUM family were down-regulated under stress and, with certain exceptions, were not responding to salt shock. On the other hand, COBRA-7 was up-regulated under shock and silenced under stress exclusively in sugar beet.

#### Pathogen response

Hypersensitive-induced response 1 was up-regulated by salt regardless of the mode of application. On the other hand, genes encoding chitin-degrading and ribosome-inactivating proteins were generally silenced, but under shock particular chitinase-encoding transcripts were induced. Conversely, osmotin was induced under stress only. Certain pathogen response-related gene families displayed complex behavior. Hfr-2 family ones were down-regulated under shock, whereas under stress a single transcript was silenced in sea beet, and two were induced in sugar beet. Among defensins, majority was down-regulated under shock, and in sugar beet under stress, while a single transcript was induced in sea beet under stress. Two remorins responded to shock with up-regulation, while one transcript was silenced. Thaumatin and RPP13–1 were specifically induced under stress, in sea- and sugar beet, respectively. Snakin-2 was up-regulated under shock exclusively in sea beet.

#### Photosynthesis

Genes encoding photosynthesis-involved proteins were down-regulated, regardless of salt treatment type and genotype. Particularly, RUBISCO small subunit and light-harvesting chlorophyll a-b binding (LHCB) protein genes were silenced under stress, while LHCBs and photosystem proteins were down-regulated under shock.

#### Secondary metabolism

Cytochrome P450 genes were generally up-regulated under shock, with certain exceptions, while a few transcripts were silenced under stress. Conversely, putative codeinone reductase was up-regulated under stress and down-regulated under shock.

#### Protein processing

Ubiquitin expression was increased in both genotypes by stress. Genes coding other proteasome components were generally down-regulated in sugar beet under stress, while under shock roughly equal numbers of transcripts were induced and silenced.

#### Nitrogen management

Out of four bark storage protein-coding DEGs, two were up-regulated by stress in sugar beet, of which one variant was induced very strongly and in a dose-dependent manner. Most of transcripts coding for NRT1/PTR FAMILY (NPF) proteins were up-regulated in leaves of stressed plants and predominantly down-regulated under shock.

#### Other genes

Salt induced changes in expression of other genes not belonging to the categories described above. Importantly, two transcripts coding for proteins involved in long distant transport were strongly up-regulated under salt stress in both genotypes. These were phloem filament protein PP1 and ureide permease transcript.

### Putative bHLH binding sites are more frequent in promoter regions of salt-regulated genes than in non-regulated ones

Prompted by its high expression level and salt-dependence in both stress and shock treatments, we analyzed bHLH137 sequence in more detail. The gene is located on chromosome 5 (Bv5_111360_mzap), has 6108 bp and encodes two transcripts that give rise to proteins of 271 and 272 aa. We found only the former in our data. Bv5_111360_mzap promoter region (defined as 1500 bp upstream of transcription start site) contains 10 binding sites for bHLH, 33 sites for WRKY, 19 for NAC, 30 for MYB and 48 for bZIP TFs. bHLH137 protein clearly belonged to bHLH family with its characteristic motifs (basic site and helix-loop-helix, Additional file 1: Figure S5). Its closest homologs comprised respective genes from *Chenopodiaceae* plants, such as spinach or *Chenopodium*, with other dicots being more distant (Additional file 1: Figure S6). The protein is also related to other subfamilies of the bHLH family, e.g. to bHLH57. bHLH canonical binding sites (E-boxes) were significantly more frequent (two tailed Fisher’s exact test, OR = 8.94, *p*-value < 0.01) in promoter regions of DEGs identified in our study than in promoters of non-DEGs.

## Discussion

Our study was aimed at a comparison of salt response in sugar beet, represented by one of typical cultivars, and its wild, halophytic ancestor – sea beet. The ‘Huzar’ cultivar was chosen as i) it is representative for contemporary sugar beet cultivars in terms of productivity traits and ii) as we found that it differed from *B. maritima* in terms of molecular mechanisms of salt response [12, 13]. However, one has to bear in mind that significant differences occur among beet cultivars, involving traits engaged in salt acclimation. Therefore we expect that, while the set of acclimation mechanisms should be universal, the importance of a given mechanism in a given cultivar may be different than in the one we studied.

Analysis of transcriptomic alterations under salt stress and salt shock may provide insight to different aspects of salt response. The analysis of leaves of stressed plants reveals transcripts involved in acclimation to salinity, i.e. altered in their expression under the control of the mechanisms limiting salt influx. On the other hand, due to its immediate nature, shock treatment may reveal transcripts involved in an ‘emergency response’, when there is no possibility of acclimation. Both kinds of salt response may be affected by genetic factors, so it is justified to study them in both genotypes.

Similar acclimation responses were visible both in sugar and sea beet. The lowered transpiration observed under stress was probably due to higher levels of ABA causing closing of stomatal apparatuses. This observation, together with lower water content and reduced leaf area (which was due to smaller mesophyll cells) observed in sugar beet as well as decreased photosynthetic rate, point at the water saving and anabolic processes deceleration strategy adopted by stressed plants, as reported previously [22, 23]. Certain traits involved in sugar beet acclimation (reduction of leaf size, accumulation of osmoprotectants) reflected sea beets adaptation to saline habitats.

Under shock the acute effects of salinity were maximized, and osmotic adjustment occurred, as indicated by increased proline, sodium, chloride and RWC. Such an effect was observed previously in beet [24] as well as in many other plants, e.g. [25–27] (Additional file 1: Figure S1, S2 and S3).

At the cellular level, the results of indirect transcription and translation measurements suggested that salt acclimation required deeper changes in cellular functions in the domesticated plant than in the wild one, showing adaptation of see the beet to salinity. The changes were more pronounced in leaves under shock, which was probably caused by uncontrolled salt influx. The increase in polyA RNA level in the sea beet under shock (Fig. 5d) might be explained by detection of 3′ polyadenylated ends of unprocessed/undegraded transcripts accumulated due to the lowered translational capacity (Fig. 5f). Contrastingly, in sugar beet, the decrease in polyA RNA levels might result from their efficient processing by translational machinery, as it was found that efficient translation might be connected with mRNA degradation [28].

Due to different life cycles and habitats, it may be expected that different traits are expressed in unstressed sugar and sea beet. Here, we show that constitutive expression of pathogen response genes distinguish wild and crop beet suggesting sea beet’s adaptation to biotic stress. On the other hand, the sugar beet was bred to increase the biomass and taproot sugar content, which required larger leaf size enabling more intensive photosynthesis, and consequently larger cell size. This trait is hallmarked by concerted high expression of enzymes increasing cell wall plasticity (Additional file 1: Table S2) [29, 30].

The differences between genotypes were deepened by salt treatments. Certain DEGs were characteristic for one of the two genotypes. We postulate that DEGs occurring solely in sugar beet may represent traits acquired during domestication, those found exclusively in the sea beet would be lost during domestication, while those common to the two subspecies would be inherited by the sugar beet from its wild ancestor. The ability to decrease leaf and cell size in response to salt stress may be a trait acquired during domestication. It requires many genes to be silenced in order to restrict growth and conserve energy and resources. On the other hand, the ability to maintain high proline and betaine levels under stress seems to have been lost by the crop. Traits inherited by the sugar beet from its wild ancestor comprise: i) photosynthesis inhibition, ii) utilization of ‘cheap osmoprotectants’, iii) wax and cuticle deposition and iv) nitrogen management regulation.

Beet leaves transcriptome rearrangement upon salt treatment was more broad-scaled under shock than under stress (Figs. 6, 7). This is not surprising, as in the case of shock, the rapid nature of the treatment is further exacerbated by facilitated salt penetration into the tissues. In the present article we partially recapitulate experimental setup published earlier [24], and the results were largely concordant. However, the broader scope of the present project, involving sugar beet, and greater sequencing coverage as well as improved replication allowed identification of sugar beet-specific and rare transcripts that could not have been detected previously. The rare transcripts were involved mainly in gene expression regulation, e.g. bHLH137. On the other hand, in the present experiments we did not observed neither differential expression of genes involved in ribosome structure and function, such as ribosomal proteins nor the ones encoding anti-oxidant proteins. This might have been caused by a difference in experimental setup. Previously we used continuous irradiation, which in combination with salinity might have affected expression of these genes, due to the constant light posing additional stress to plants. To avoid this stress we applied regulated photoperiod in the present study.

The magnitude of transcriptomic changes under shock was similar for both genotypes, and largely the same genes were differentially expressed. There was no single GO category specific for the sugar beet, instead DEGs were scattered among a few of them. The increased expression of transcripts involved in betaine and proline synthesis may contribute to the osmotic adjustment in salt-stressed leaves of *B. maritima*. Conversely, the higher P5CDH expression may restrict proline accumulation in leaves of salt stressed plants [31]. The regulation of proline level may be important under long term salt treatments since excessive levels are toxic to plant cells [32, 33]. Remarkably, P5CDH expression decreased under salt shock conditions, which, together with concomitant increase of P5Cs level, might have caused the proline level to be much higher than under salt stress (Additional file 1: Table S3).

Among DEGs encoding proteins involved in membrane transport aquaporins, ion channels and sugar transporters were prevailing (Additional file 1: Table S3). Under salt stress, aquaporin down-regulation may facilitate water saving as demonstrated by Afzal et al. [34] and Li et al. [35] and decrease in ion channels expression may limit toxic ions influx [36]. On the other hand, their increased expression in salt-shocked leaves may contribute to ‘osmotic recovery’, as demonstrated previously [24].

Besides osmotic adjustment, water saving is another important part of plant long-term response to salinity [37]. Wax deposition and cuticle synthesis efficiently contribute to limiting water losses due to transpiration [38, 39]. Accordingly, we observed lowered transpiration under salt stress. This decrease might be, at least partially, attributed to the observed increased level of lipid transfer proteins-encoding transcripts involved in the aforementioned processes [40]. Similarly, under shock, an increased ECERIFERUM transcript level might contribute to increased wax synthesis. This transcript encodes very-long-chain fatty acids (VLCFA) synthase engaged in this process [41]. The increase in the expression of ECERIFERUM gene in response to salinity and drought has been reported for *Chrysopogon zizanioides*, a perennial C4 grass, tolerant to water, salinity and submergence stress [41].

Silenced genes dominated among DEGs under salt stress (Additional file 1: Table S3). Such an effect was frequently observed in transcriptomic experiments on plants under abiotic stress [42, 43] and might be explained by energy and resources conservation strategy adopted by stressed plants. In the case of beet, this silencing was more pronounced in the sugar beet, which might have been caused by the necessity to down-regulate constitutively expressed genes involved in growth and productivity traits. This large-scale down-regulation was probably mediated by the concerted decrease in expression of many transcription factors, which was not observed in the sea beet. We observed only one transcription factor whose expression was greater both under stress and shock, namely bHLH137 (Additional file 1: Table S3). This is the first observation implying its being the key TF in salinity response. It belongs to a large family of TFs frequently implied in abiotic stress response [44–46]. It seems plausible that it is NaCl-inducible, as indicated by its salt dose-dependent expression changes, and that it may play the key role in activation of salt-induced genes. This view is supported by significantly more frequent incidence of bHLH binding sites (E-boxes) in promoter regions of DEGs than promoters of non-DEGs. However, further experimental studies would be required to confirm its role in salt response.

For the first time we identify nitrogen management genes as involved in salt response in beet (Additional file 1: Table S3). NRT family proteins participate in nitrogen uptake [47], while bark storage proteins (BSPs) are involved in seasonal and short term nitrogen storage [48]. The role of BSPs in non-woody plants remains unclear, however, in the case of the sea beet, their high expression might be an adaptation to rapidly changing environmental conditions, involving nitrogen limitation due to salinity. This limitation might be a consequence of restricted nutrients uptake from salt-affected soil [49]. Consistently with this view, an increase of BSPs expression to levels comparable with those in the sea beet was observed in sugar beet under salt stress (Additional file 1: Table S2).

## Conclusions

Our results suggest that maintaining homeostasis under salt stress requires deeper transcriptomic changes in the sugar beet than in the sea beet. Mass genes down-regulation due to salt stress may be attributed to the silencing of genes encoding numerous transcription factors and signaling proteins, which results in restricted growth and certain aspects of metabolism. Concomitantly, the up-regulation of lipid transfer protein-encoding genes suggests that increased wax deposition may be involved in lowering water loss under stress. The up-regulation of genes encoding NRTs and bark storage proteins suggests their new, yet undefined, role in beet acclimation to salinity. In both genotypes salt shock elicits greater changes in gene expression pattern than stress and it results in greater number of up-regulated genes compared to the latter. The majority of activated genes participates in osmotic adjustment. The dose-dependent salt inducibility of bHLH137 and the greater incidence of bHLH-binding motives in promoter regions of salinity-regulated genes makes the gene a putative key regulator of salinity response.

## Methods

### Plant material

*B. maritima* (seeds obtained from National Germplasm Resources Laboratory, Beltsville, MD, USA), and sugar beet cv. ‘Huzar’ (WHBC Poznań, Poland) were used as a plant material. The seeds (30 per pot) were sown into 40 × 16 × 14 (l × w × d) cm pots filled with sand and vermiculite (1/1 *v*/v) and plants were watered regularly with half-strength Hoagland solution. Plant culture and subsequent treatments with salinity were performed in a growth chamber with a photoperiod of 16 h of light and 8 h of darkness with standard irradiation of 30 ± 5 μmol m^− 2^ s^− 1^, provided by T8 15 W 6500 K “Daylight” tubes (POLAMP, Poland). The temperature regime was 25 °C during the day and 18 °C in the night.

### Exposing plants to salinity

We used two, drastically different, approaches to exposing plants to salinity: i) stress, imitating natural situation whereby changes in salinity are gradual and slow, and the salt uptake takes place via roots and ii) shock, which does not occur in nature, but it allows to study leaf cells salt response uninfluenced by mechanisms restricting salt delivery to leaves.

Salt stress. Potted plants were subjected to either moderate (150 mM NaCl) or strong (300 mM NaCl) stress during 32-day-long period. Salt treatments started when the first pair of mature leaves fully developed. Over the first 16 days of treatment, plants were watered in two-day-long intervals, with half-strength Hoagland medium supplemented with increasing concentrations of NaCl, until the final concentrations of 150 mM and 300 mM were reached, then the treatments were continued for 16 days. Untreated controls were watered with unsupplemented medium. Plants were watered with 200 ml of solution per pot containing 2 l of growth substrate. Leaves representing the second pair of true leaves, were collected for molecular analysis. Separate plants grown in parallel were used for morphological, cytological, physiological and biochemical analyses.

#### Salt shock

Fully developed second true leaves were excised, cut to the same petiole length, and subjected to salt shock as described by Skorupa et al. [24].

### Experimental design

**Fig. 8:**
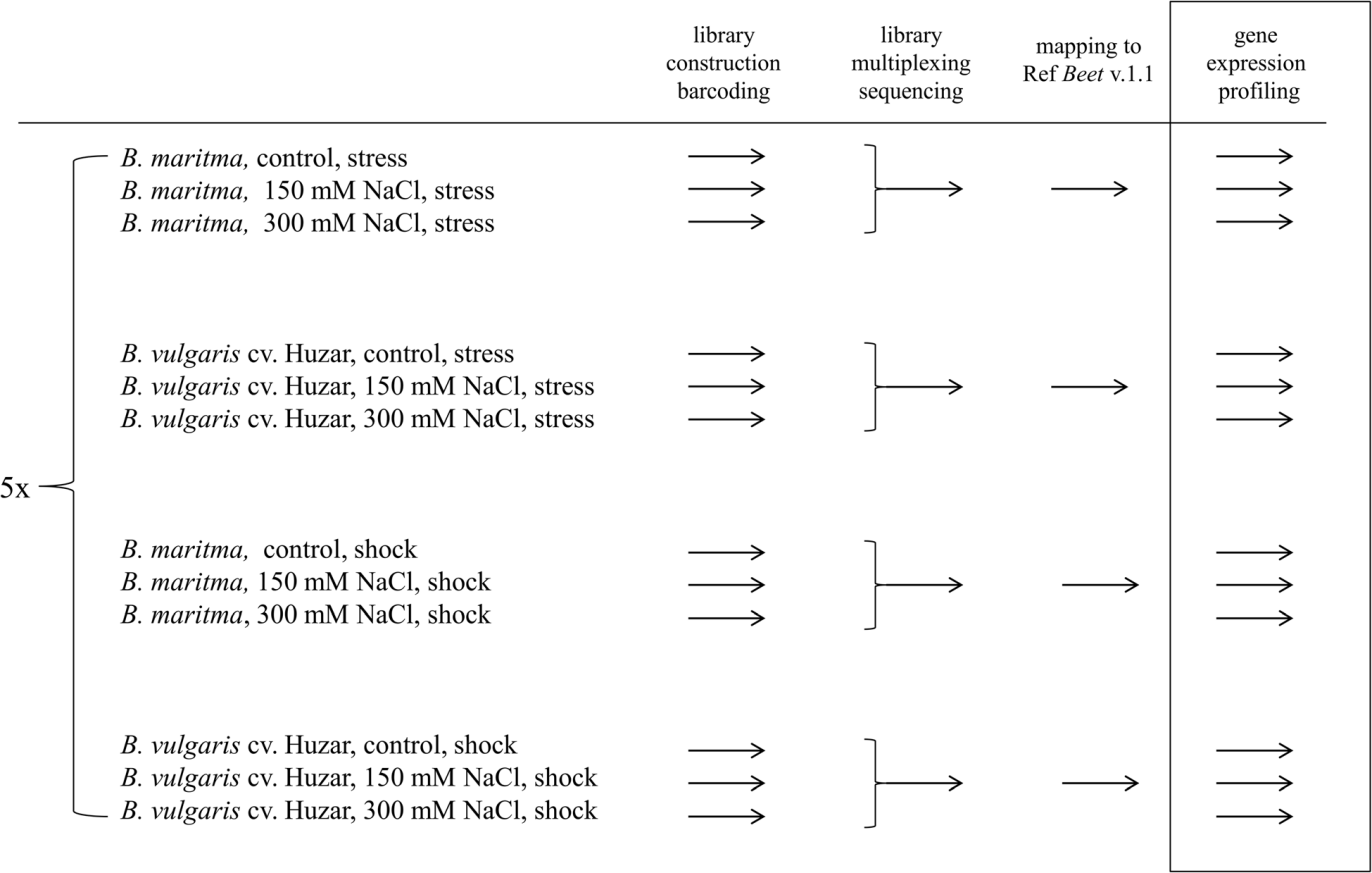
Outline of the transctiptomic experiment

For determination of morphological, physiological, cellular, biochemical and chemical parameters we used three biological replicates consisting of 30 plants per treatment. The measurements were performed on 10 fully expanded leaves from each experimental variant. For transcriptomic analysis, twelve combinations of genotype, mode of salt treatment and salt concentration were prepared (Fig. 8). For each combination, five biological replicates (individual plants) were used.

### RNA isolation, sequencing libraries preparation, quantification and sequencing

Total RNA was isolated from leaves using TriPure reagent (Roche) and digested with DNase I (Thermo Scientific). After RNA quality and quantity analysis, one library was generated for each of the biological replicates using TruSeq Stranded Total RNA Sample Preparation Kit with Ribo-Zero Plant (Illumina) (details in Additional file 2) and sequenced on MiSeq using 2 × 75 cycles kit v.3 (Illumina). Bioinformatic analysis was performed as described previously [24], but the reads were mapped to RefBeet v.1.1 genomic sequence [14] instead of the Trinity-assembled transcriptome. Detailed description of processing and analysis of the RNASeq data is provided in Additional file 2. Raw reads are available in NCBI SRA under BioProject PRJNA453103.

### Experimental validation of differential expression data by qRT-PCR

Gene expression patterns of selected transcripts were validated by real time qPCR analysis as described earlier [24]. Further details are provided in Additional file 2.

### Fluorescence in situ hybridisation (FISH) of polyA RNA and 25S rRNA and cell volume measurements

Leaves were processed according to modified protocol of Tirichine et al. [50]. Cell volume was assessed as a sum of areas of all optical sections coming from a given cell multiplied by Z-axis step. FISH detection of polyA RNA and rRNA was performed according to Dełeńko et al. [51] with modifications. For further details see Additional file 2.

### Determination of biochemical, physiological and morphological parameters

Chlorophyll and proline were estimated according to Witham et al. [52] and Abrahám et al. [53], respectively. Abscisic acid (ABA) was extracted and quatified by chromatography/mass spectrometry according to modified protocol of Vine et al. [54]. Detailed protocols are provided in Additional file 2. Leaf gas exchange, chlorophyll content index (CCI), relative water content (RWC), fresh weight (FW) of the above-ground plant part, roots length as well as leaves surface and number were determined as described in Additional file 2.

### Determination of plants mineral content

P, K, Mg, Na, Ca, Fe, Mn, Cu, Zn, B, Cl as well as N (as NH_3_) content was determined as described in Additional file 2.

### Statistics

For morphological, physiological, cellular, biochemical and chemical parameters statistical significance of differences between means was determined with ROBUST ANOVA (raov of the rfit R package, [55] When the general test was significant, Tukey’s HSD test was run on ANOVA results (aov and TukeyHSD in R). Differences with *p* < 0.01 were considered significant. Error bars shown in all figures represent standard deviation calculated from all repetitions of each experiment.

Significance of differences in bHLH binding site incidence between non-DEGs and DEGs sets was assessed with two-tailed Fisher’s exact test implemented in R.

Significance of differences in transcripts levels was assessed with Cuffdiff [56], and the false discovery rate (FDR) q < 0.01 was regarded significant. GO enrichment was performed with topGO R package [57], for details see Supplementary Methods. nMDS (metaMDS) and PERMANOVA (adonis) analyses were performed on Morisita-Horn [58] distance matrix (vegdist) (vegan R package, [59] obtained from Wisconsin-transformed differentially expressed genes count matrix. *P* < 0.01 was considered significant.

## Additional files

Additional file 1: Supplementary Figures and Tables. (DOCX 13960 kb)

Additional file 2: Supplementary Methods. (DOC 97 kb)

Additional file 3: Expression levels (in FPKM) for all transcripts identified in the study. (XLSX 10168 kb)

Additional file 4: Changes in expression levels of DEGs responding to salt stress or shock and assigned to ‘COMMON’ category. ‘COMMON’ – genes that were differentially expressed both in sea- and sugar beet. MC - sea beet (*B. maritima*), control; M150 - sea beet (*B. maritima*), 150 mM NaCl; M300 - sea beet (*B. maritima*), 300 mM NaCl; HC - sugar beet (*B. vulgaris* cv. ‘Huzar’), control; H150 - sugar beet (*B. vulgaris* cv. ‘Huzar’), 150 mM NaCl; H300 - sugar beet (*B. vulgaris* cv. ‘Huzar’), 300 mM NaCl. (XLS 302 kb)

Additional file 5: Changes in expression levels of DEGs responding to salt stress or shock and assigned ‘UNIQUE’ category. ‘UNIQUE’ – genes that were differentially expressed in one of the genotypes only. MC - sea beet (*B. maritima*), control; M150 - sea beet (*B. maritima*), 150 mM NaCl; M300 - sea beet (*B. maritima*), 300 mM NaCl; HC - sugar beet (*B. vulgaris* cv. ‘Huzar’), control; H150 - sugar beet (*B. vulgaris* cv. ‘Huzar’), 150 mM NaCl; H300 - sugar beet (*B. vulgaris* cv. ‘Huzar’), 300 mM NaCl. (XLS 246 kb)

## Abbreviations

ABA: Abscisic acid
AFU: Arbitrary fluorescence unit
BAC: Bacterial artificial chromosome
BADH: Betaine aldehyde dehydrogenase
bHLH: Basic helix-loop-helix proteins
BSP: Bark storage proteins
CCI: Chlorophyll content index
DEG: Differentially expressed gene
EST: Expressed sequence tag
FISH: Fluorescence in situ hybridization
FPKM: Fragments Per Kilobase Million
FW: Fresh weight
GO: Gene Ontology
h: Hours
P5CS: δ-1-pyrroline-5-carboxylate synthase
PP1: Protein phosphatase 1
RPP: Resistance to *Peronospora parasitica*
RUBISCO: Ribulose bisphosphate carboxylase-oxygenase; LHCB: light-harvesting chlorophyll a-b binding
TF: Transcription factor
VLCFA: Very-long-chain fatty acids
