# Supplementary material for: Salt stress vs. salt shock - the case of sugar beet and its halophytic ancestor
Source: BMC Plant Biol. 2019 Feb 6;19:57. doi: 10.1186/s12870-019-1661-x (PMC6364445; doi:10.1186/s12870-019-1661-x)
Supplement: Supplementary file 1 — Supplementary Figures and Tables. (DOCX 13960 kb) [file 12870_2019_1661_MOESM1_ESM.docx]

**Salt stress vs. salt shock - the case of sugar beet and its halophytic ancestor**

Monika Skorupa^1*^, Marcin Gołębiewski^1,2*^, Katarzyna Kurnik^2^, Janusz Niedojadło^3^, Jacek Kęsy^2^, Krzysztof Klamkowski^4^, Katarzyna Wójcik^4^, Waldemar Treder^4^, Andrzej Tretyn^1,2^, Jarosław Tyburski^1,2*#^

^1^ Centre for Modern Interdisciplinary Technologies, Nicolaus Copernicus University, Toruń, Poland; ^2^ Chair of Plant Physiology and Biotechnology, Faculty of Biology and Environment Protection, Nicolaus Copernicus University, Toruń, Poland; ^3^ Department of Cell Biology, Faculty of Biology and Environment Protection, Nicolaus Copernicus University, Toruń, Poland; ^4^ Research Institute of Horticulture, Skierniewice, Poland

*** -** these authors contributed equally to this work

# - corresponding author, addresses:

Author for correspondence:

*Jarosław Tyburski*

*Tel: +48 56 611 47 73*

*Email:* [*tybr@biol.umk.pl*](mailto:tybr@biol.umk.pl)





**Fig. S1.** Morphological and physiological parameters determined for sea- (*B. maritima*) and sugar beet (*B. vulgaris cv.* 'Huzar') subjected to salt stress (a,b) or shock (c,d). Roots length (a), chlorophyll content index (CCI; b,d), transpiration rate (c). Numbers above the bars indicate fold changes relative to control. Different letters above the bars indicate significant differences at p < 0.01 (ANOVA followed by Tukey’s test).


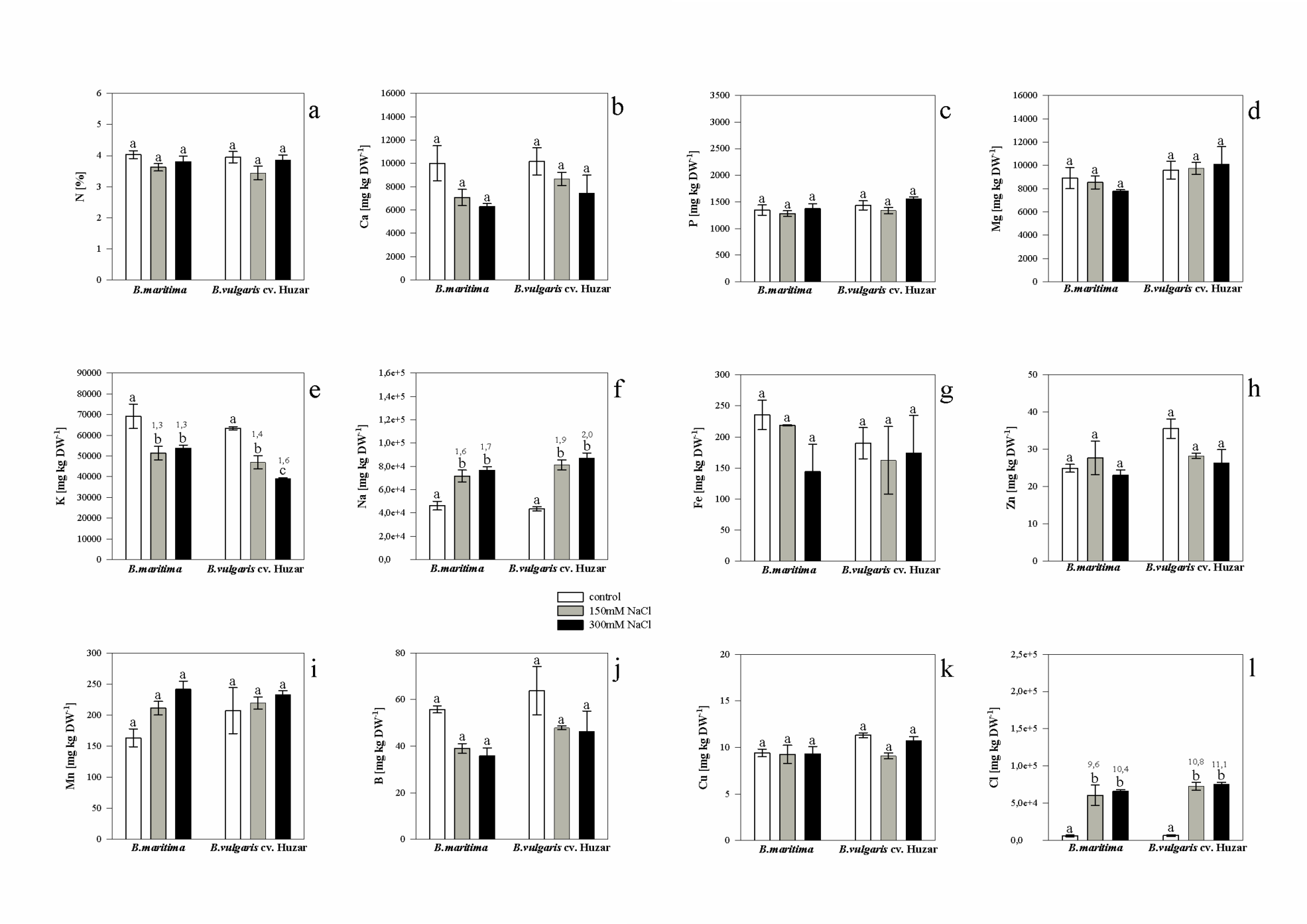


**Fig. S2**. Leaf mineral composition determined for sea- (*B. maritima*) and sugar beet (*B. vulgaris* cv. 'Huzar') subjected to salt stress. N (a), Ca (b), P (c), Mg (d), K (e), Na (f), Fe (g), Zn (h), Mn (i), B (j), Cu (k) and Cl (l). Numbers above the bars indicate fold changes relative to control. Different letters above bars indicate significant differences at p < 0.01 (ANOVA followed by Tukey’s test).


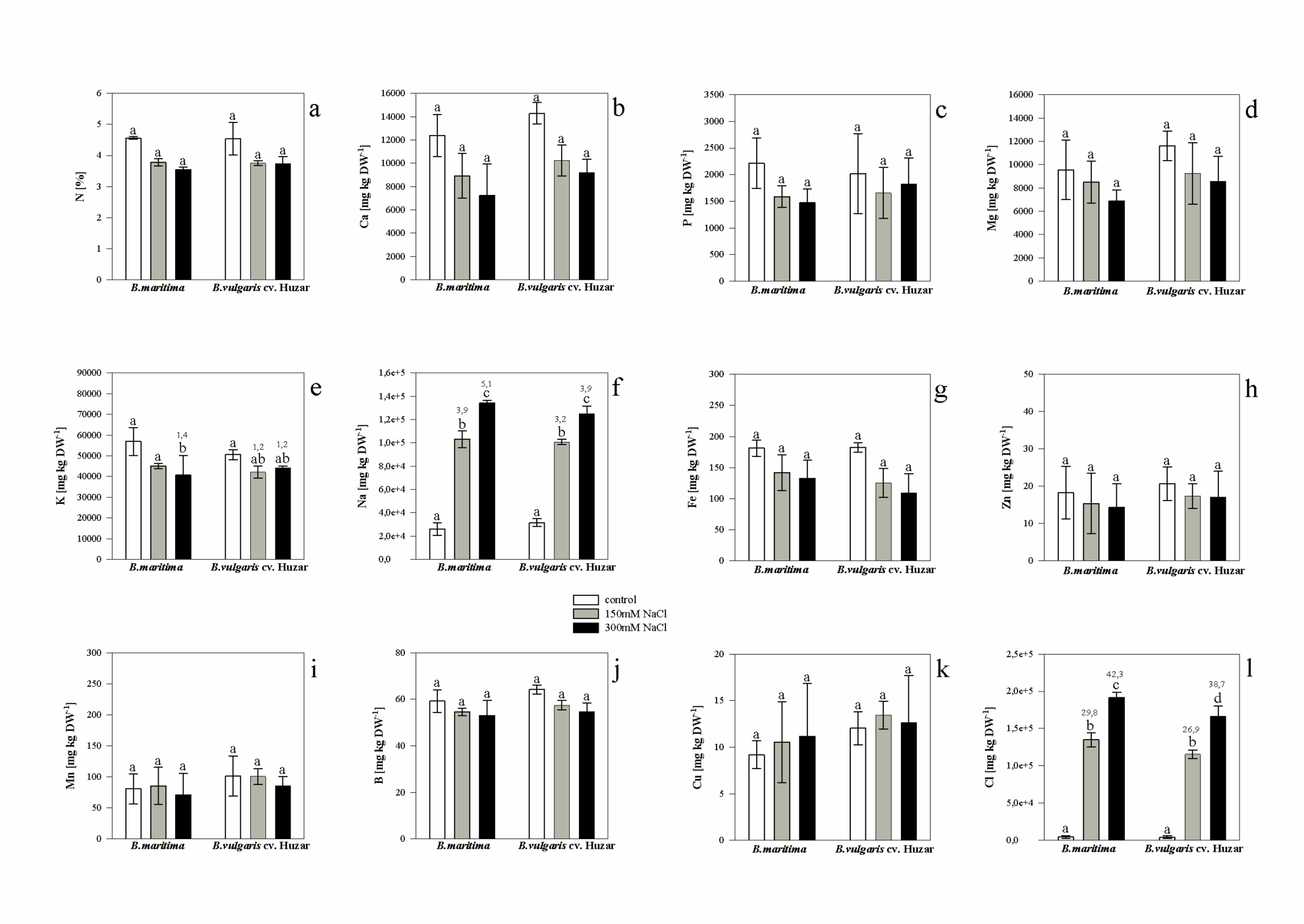


**Fig. S3.** Leaf mineral composition determined for sea- (*B. maritima*) and sugar beet (*B. vulgaris cv.* 'Huzar') subjected to salt shock. N (a), Ca (b), P (c), Mg (d), K (e), Na (f), Fe (g), Zn (h), Mn (i), B (j), Cu (k) and Cl (l). Numbers above the bars indicate fold changes relative to control. Different letters above bars indicate significant differences at p < 0.01 (ANOVA followed by Tukey’s test).





**Fig. S4**. Physiochemical parameters of growth substrate solution determined after salt stress treatments. pH, EC (a), N-NO_3_, P, K, Mg (b), Ca, Na, Cl (c). Different letters above bars indicate significant differences at p < 0.01 (ANOVA followed by Tukey’s test).


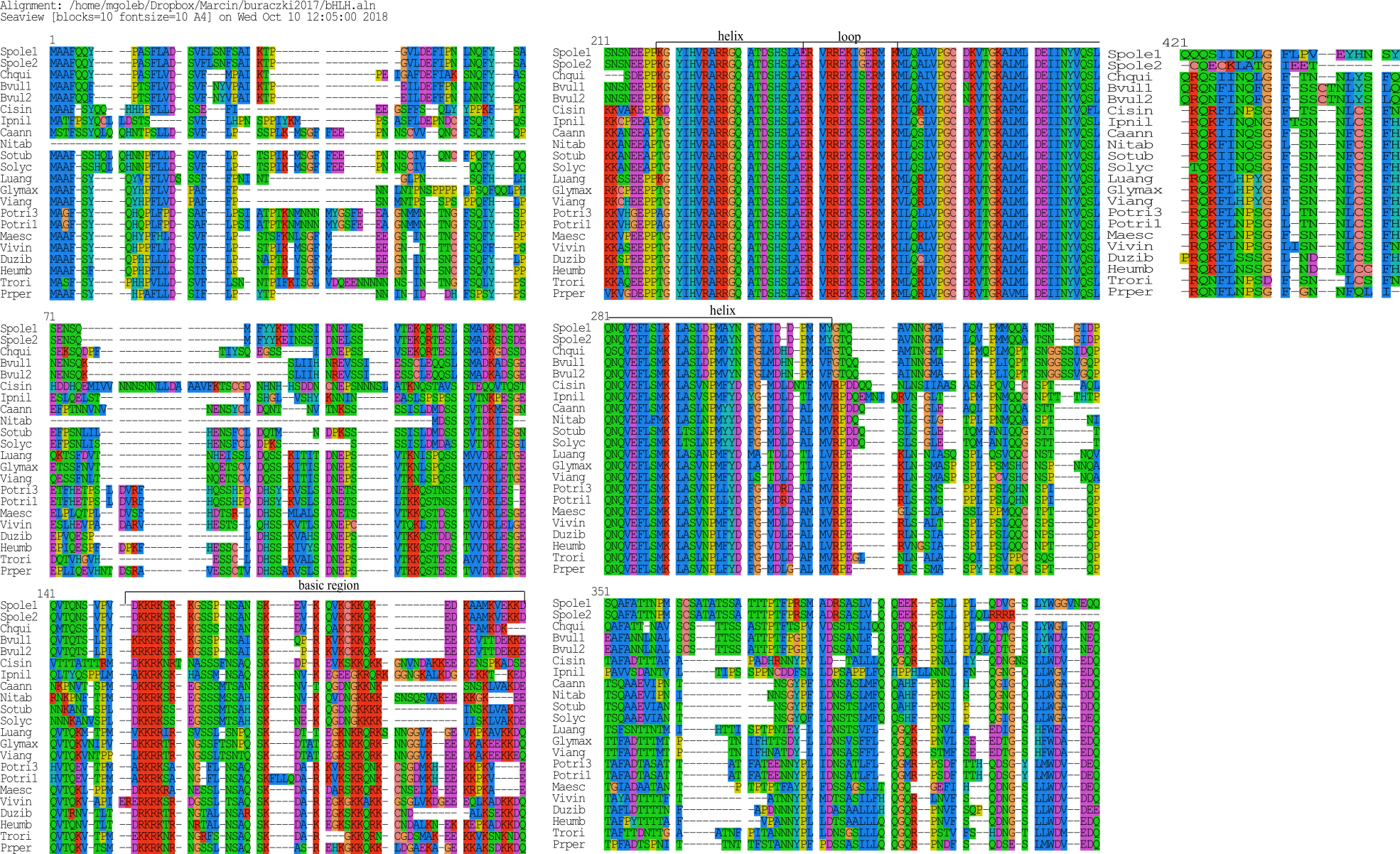


**Fig. S5.** Alignment of the deduced amino acid sequences of bHLHs from selected plant species. Abbreviations denote: Bvvul – *Beta* *vulgaris*; Chqui – *Chenopodium* *quinoa*; Spole – *Spinacia* *oleracea*; Ipnil – *Ipoёma* *nil*; Nitab – *Nicotiana* *tabacum*; Caann – *Capsicum* *annuum*; Sotub – *Solanum* *tuberosum*; Solyc – *Solanum* *lycopersicum*; Vivin – *Vitis* *vinifera*; Duzib – *Durio* *zibeticum*; Heumb – *Herrania* *umbratica*; Moesc – *Manihot* *esculenta*; Potri – *Populus* *trichocarpa*; Cisin – *Citrus* *sinensis*; Luang – *Lupinus* *angustifolium*; Glymax – *Glycine* *max*; Viang – *Vigna* *angularis*; Trori – *Trema* *orientalis*; Prper – *Prunus* *persica*


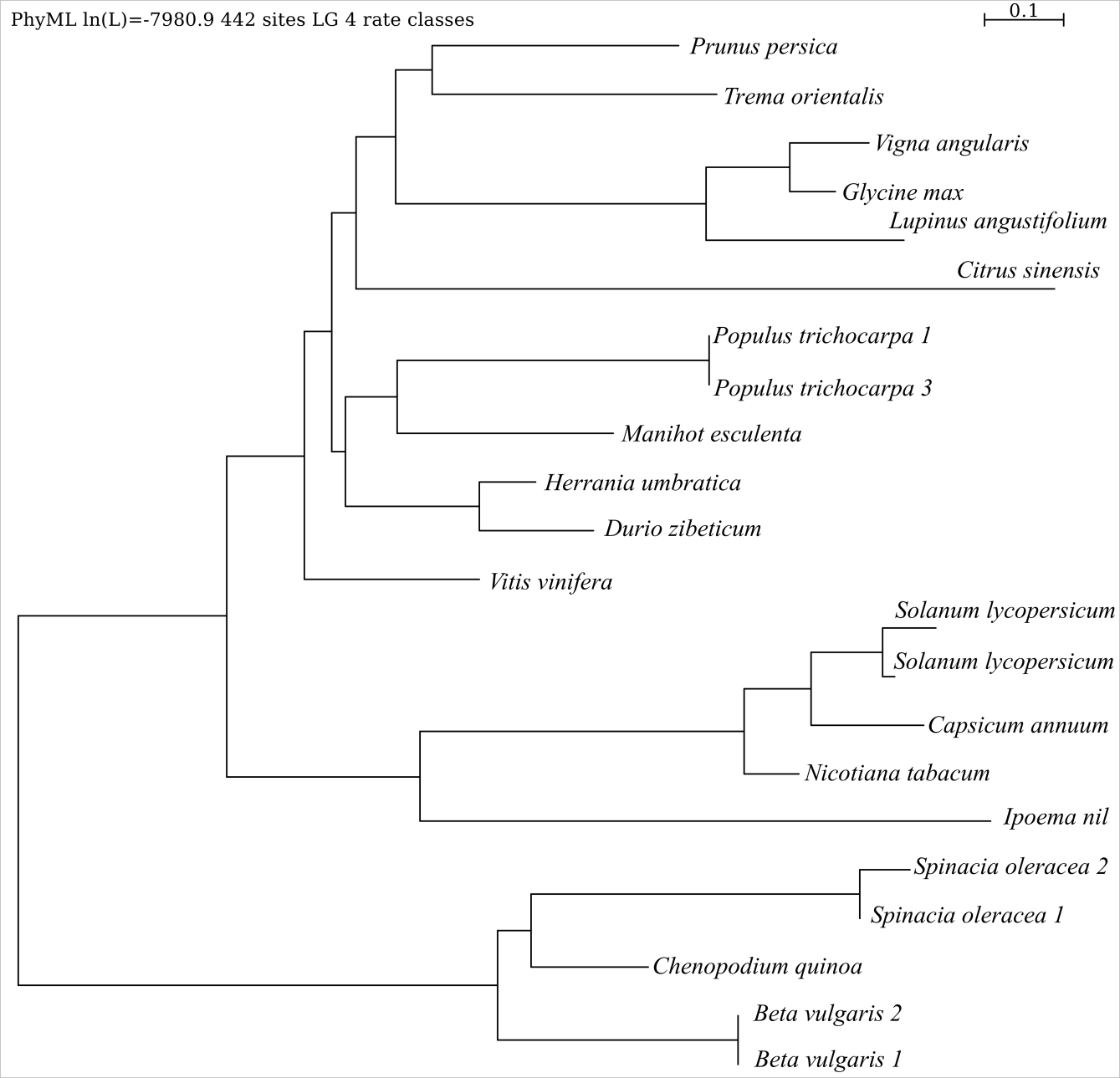


**Fig. S6.** Maximal likelihood phylogenetic tree of bHLH137 proteins obtained with Phyml.

**Table S1**. Sequencing statistics.

|  | Mode | Total no. of reads | Average no. of reads (±SD) | Mapped left reads (%) | Mapped right reads (%) |
| --- | --- | --- | --- | --- | --- |
| *B. maritima* control | stress | 37 044 312 | 7 408 862 (±3 706 488) | 74.6% | 72.0% |
| *B. maritima* 150 mM NaCl |  | 42 392 528 | 8 478 506 (±5 015 008) | 79.8% | 75.6% |
| *B. maritima* 300 mM NaCl |  | 35 460 370 | 7 092 074 (±1 975 081) | 77.5% | 74.5% |
| *B. vulgaris* cv 'Huzar' control |  | 48 452 434 | 9 690 487 (±3 400 435) | 77.9% | 75.6% |
| *B. vulgaris* cv 'Huzar' 150 mM NaCl |  | 35 713 478 | 7 142 696 (±3 905 612) | 76.6% | 73.3% |
| *B. vulgaris* cv 'Huzar' 300 mM NaCl |  | 41 894 466 | 8 378 893 (±3 592 159) | 78.4% | 75.4% |
| *B. maritima* control | shock | 26 470 214 | 5 294 043 (±983 067) | 73.7% | 73.1% |
| *B. maritima* 150 mM NaCl |  | 33 283 704 | 6 656 741 (±1 024 677) | 73.6% | 72.6% |
| *B. maritima* 300 mM NaCl |  | 23 964 546 | 4 792 909 (±1 282 587) | 77.8% | 76.2% |
| *B. vulgaris* cv 'Huzar' control |  | 33 984 462 | 6 796 892 (±711 581) | 75.9% | 74.8% |
| *B. vulgaris* cv 'Huzar' 150 mM NaCl |  | 24 994 980 | 9 998 996 (±767 550) | 70.6% | 69.9% |
| *B. vulgaris* cv 'Huzar' 300 mM NaCl |  | 29 733 644 | 5 946 789 (±1 643 333) | 71.9% | 71.7% |

**Table S2.** Expression levels (in FPKM) of genes that were differentially expressed in leaves of unstressed plants. FPKM – Fragments Per Kilobase of transcript and Million reads.

|  | | | *Beta maritima* | | | | | |
| --- | --- | --- | --- | --- | --- | --- | --- | --- |
| Id | **name** | **function** | **FPKM** | | | | | |
|  |  |  | control | | 150 mM NaCl | | 300 mM NaCl | |
|  |  |  | Mar | Huz | Mar | Huz | Mar | Huz |
| XLOC_004426 | cellulose synthase | cell wall plasticity | 17,11 | 3,12 | 13,88 | 2,22 | 8,73 | 2,29 |
| XLOC_023390 | expansin | cell wall plasticity | 23,41 | 4,67 | 16,99 | 5,92 | 13,55 | 10,54 |
| XLOC_024953 | pectate lyase | cell wall plasticity | 5,59 | 1,20 | 3,08 | 1,21 | 1,49 | 0,79 |
| XLOC_033267 | beta-glucosidase 12 | cell wall plasticity | 181,54 | 18,65 | 28,39 | 16,61 | 11,83 | 6,00 |
| XLOC_055110 | proline-rich 4 protein | cell wall plasticity | 13,09 | 1,27 | 5,53 | 2,58 | 1,19 | 0,28 |
| XLOC_007448 | chitinase | patogen response | 18,39 | 3,79 | 1,38 | 4,35 | 0,29 | 0,36 |
| XLOC_014663 | disease resistance RPP13 1 protein | patogen response | 11,54 | 2,79 | 14,86 | 27,95 | 15,23 | 18,13 |
| XLOC_016532 | cytosolic sulfotransferase 5 | patogen response | 10,21 | 1,66 | 6,67 | 2,01 | 2,44 | 2,33 |
| XLOC_020157 | jasmonate-induced protein | patogen response | 1662,13 | 129,73 | 1789,61 | 210,01 | 1067,61 | 118,32 |
| XLOC_024669 | jasmonate-induced protein | patogen response | 59,84 | 6,40 | 49,59 | 29,84 | 51,39 | 46,82 |
| XLOC_051409 | jasmonate-induced protein | patogen response | 21,89 | 0,00 | 21,39 | 1,33 | 12,49 | 0,00 |
| XLOC_021594 | vinorine synthase | patogen response | 5,03 | 0,96 | 2,98 | 1,48 | 1,08 | 1,24 |
| XLOC_025621 | miraculin | patogen response | 11,19 | 2,62 | 4,41 | 6,11 | 2,77 | 2,59 |
| XLOC_027579 | glucan endo-1,3-beta-glucosidase | patogen response | 22,05 | 4,51 | 16,79 | 7,57 | 17,88 | 4,17 |
| XLOC_027796 | terpene synthase | patogen response | 95,03 | 9,48 | 69,98 | 14,54 | 34,82 | 28,92 |
| XLOC_034905 | defensin J1-2 | patogen response | 115,33 | 27,66 | 256,61 | 47,40 | 316,91 | 53,13 |
| XLOC_035412 | inhibitor of trypsin and hageman factor | patogen response | 662,30 | 107,89 | 153,20 | 221,07 | 92,17 | 90,22 |
| XLOC_037415 | polyphenol chloroplastic | patogen response | 65,29 | 16,16 | 13,95 | 15,57 | 9,16 | 9,58 |
| XLOC_037416 | polyphenol chloroplastic | patogen response | 13,89 | 1,17 | 0,23 | 2,42 | 0,57 | 5,61 |
| XLOC_037644 | alkane hydroxylase MAH1 | patogen response | 14,58 | 3,14 | 7,33 | 1,96 | 3,15 | 1,50 |
| XLOC_037958 | alkane hydroxylase MAH1 | patogen response | 5,24 | 0,91 | 4,00 | 1,72 | 1,27 | 0,81 |
| XLOC_047116 | cytochrome P450 71A26 | patogen response | 11,78 | 2,46 | 9,96 | 5,14 | 7,59 | 2,96 |
| XLOC_051155 | chitotriosidase-1 | patogen response | 10,88 | 2,60 | 9,74 | 3,58 | 11,45 | 1,77 |
| XLOC_054985 | A Chain Agglutinin in complex with T-disaccharide | patogen response | 5,75 | 0,00 | 3,37 | 0,51 | 1,25 | 0,96 |
| XLOC_056169 | O-acyltransferase WSD1 | patogen response | 8,26 | 1,29 | 17,24 | 1,84 | 6,97 | 1,48 |
| XLOC_023306 | cytosolic sulfotransferase 15 | patogen response | 7,87 | 1,50 | 5,81 | 5,03 | 4,90 | 3,86 |
| XLOC_007271 | nucleoredoxin 1 | growth regulation | 7,00 | 1,09 | 6,94 | 1,09 | 4,92 | 0,93 |
| XLOC_009789 | pentatricopeptide repeat-containing chloroplastic | growth regulation | 6,14 | 1,48 | 7,01 | 2,36 | 3,67 | 1,57 |
| XLOC_019997 | serine threonine kinase CDL1 | growth regulation | 5,15 | 1,07 | 1,63 | 0,93 | 0,98 | 0,59 |
| XLOC_021040 | vacuolar-processing enzyme | growth regulation | 6,02 | 1,37 | 2,91 | 0,73 | 3,36 | 0,53 |
| XLOC_021292 | flowering-promoting factor 1 2 | growth regulation | 27,72 | 5,23 | 4,45 | 6,84 | 2,89 | 1,86 |
| XLOC_055683 | auxin-responsive protein SAUR21 | growth regulation | 16,47 | 2,58 | 21,05 | 4,01 | 10,85 | 3,59 |
| XLOC_009010 | NEP-interacting (DUF239) | protein processing | 18,79 | 0,00 | 20,18 | 8,94 | 18,79 | 15,28 |
| XLOC_053817 | serine threonine protein kinase clkA | protein processing | 8,92 | 1,85 | 3,29 | 1,83 | 1,10 | 1,21 |
| XLOC_016258 | DNA-directed RNA polymerase I subunit 1 | nucleic acid processing | 5,13 | 0,59 | 3,64 | 0,66 | 3,91 | 0,65 |
| XLOC_024235 | probable 6-phosphogluconolactonase chloroplastic | primary metabolism | 10,03 | 1,55 | 8,74 | 3,73 | 4,31 | 2,53 |
| XLOC_023053 | inositol hexakisphosphate and diphosphoinositol-pentakisphosphate kinase 2 | signaling protein | 12,31 | 1,61 | 9,29 | 1,78 | 8,87 | 2,29 |
| XLOC_023923 | transcription factor bHLH18 | signaling protein | 14,15 | 3,12 | 9,50 | 5,27 | 6,91 | 4,69 |
| XLOC_027344 | DDT domain-containing protein PTM | signaling protein | 631461 | 111212 | 10^6^ | 321824 | 275396 | 244501 |
| XLOC_039715 | serine threonine kinase rio2 | signaling protein | 7228,41 | 670,39 | 2169,70 | 645,85 | 3467,08 | 467,04 |
| XLOC_024568 | bark storage A protein | storage protein | 255,74 | 2,43 | 575,41 | 219,86 | 933,77 | 723,63 |
| XLOC_024569 | bark storage A protein | storage protein | 876,91 | 136,26 | 1143,13 | 479,94 | 891,35 | 709,99 |
| XLOC_053207 | aquaporin PIP2-1 | transmembrane  transport | 5,35 | 0,98 | 4,28 | 1,14 | 1,45 | 0,48 |
| XLOC_066373 | maltose transporter | transmembrane transport | 9,41 | 0,00 | 2,46 | 0,00 | 2,39 | 0,00 |
|  |  |  | **Beta vulgaris cv. Huzar** | | | | | |
| XLOC_016424 | phosphate-responsive 1 family protein | abiotic stress | 1,06 | 8,78 | 0,27 | 0,89 | 0,37 | 0,85 |
| XLOC_024650 | SRC2 kinase | abiotic stress | 2,46 | 14,75 | 2,58 | 9,76 | 7,82 | 12,88 |
| XLOC_055463 | metallothiol transferase | abiotic stress | 6,28 | 44,05 | 3,49 | 3,43 | 2,56 | 11,69 |
| XLOC_015399 | mannan endo-1,4-beta-mannosidase 7 | cell wall plasticity | 0,88 | 13,06 | 1,79 | 8,62 | 1,82 | 5,91 |
| XLOC_016782 | xyloglucan endotransglycosylase hydrolase | cell wall plasticity | 1,95 | 17,25 | 0,27 | 0,89 | 1,31 | 1,63 |
| XLOC_018808 | probable xyloglucan glycosyltransferase 12 | cell wall plasticity | 1,02 | 5,19 | 0,55 | 0,73 | 0,71 | 0,79 |
| XLOC_023061 | xyloglucan endotransglycosylase hydrolase | cell wall plasticity | 1,07 | 11,82 | 0,52 | 0,49 | 0,49 | 0,50 |
| XLOC_033359 | xyloglucan endotransglucosylase hydrolase | cell wall plasticity | 4,71 | 31,09 | 1,48 | 0,24 | 0,39 | 0,27 |
| XLOC_013976 | disease resistance protein RGA3 | patogen response | 0 | 6,18 | 0,05 | 1,59 | 0,05 | 1,60 |
| XLOC_024526 | disease resistance protein RGA3 | patogen response | 0,66 | 4,93 | 1,11 | 1,51 | 1,43 | 1,19 |
| XLOC_016222 | germin 2-1 | patogen response | 2,49 | 13,15 | 2,28 | 6,82 | 3,94 | 10,31 |
| XLOC_030152 | NDR1 protein | patogen response | 2,17 | 12,75 | 2,08 | 2,58 | 2,27 | 1,87 |
| XLOC_036769 | mannose glucose-specific lectin | patogen response | 88,23 | 1022,61 | 163,60 | 658,52 | 191,07 | 284,76 |
| XLOC_048458 | ribosome-inactivating protein | patogen response | 8,20 | 50,19 | 0,43 | 4,78 | 0,59 | 1,78 |
| XLOC_052823 | YLS9 protein | patogen response | 4,10 | 36,55 | 1,91 | 0,73 | 0,93 | 2,09 |
| XLOC_016422 | EXORDIUM protein | growth regulation | 6,01 | 28,92 | 1,78 | 0,87 | 1,15 | 1,72 |
| XLOC_023895 | COBRA 7 protein | growth regulation | 2,60 | 11,64 | 1,69 | 0,97 | 0,93 | 1,44 |
| XLOC_027448 | wall-associated receptor kinase | growth regulation | 1,24 | 6,19 | 1,19 | 0,97 | 1,33 | 0,87 |
| XLOC_027450 | wall-associated receptor kinase | growth regulation | 2,43 | 11,32 | 2,26 | 11,45 | 2,53 | 7,34 |
| XLOC_031331 | abscisic acid 8 -hydroxylase 1 | growth regulation | 1,61 | 7,59 | 1,36 | 0,23 | 0,59 | 0,37 |
| XLOC_035645 | gibberellin 2-beta-dioxygenase | growth regulation | 0,81 | 7,42 | 0,48 | 0,33 | 0,09 | 0,36 |
| XLOC_036357 | probable indole-3-acetic acid-amido synthetase | growth regulation | 0,69 | 4,25 | 0,59 | 0,22 | 1,28 | 1,17 |
| XLOC_050744 | ethylene-responsive transcription factor TINY | growth regulation | 0,89 | 7,48 | 0,09 | 1,08 | 0,57 | 0,68 |
| XLOC_029127 | butyrate-CoA ligase peroxisomal | lipid metabolism | 1,34 | 24,95 | 0,78 | 0,83 | 1,09 | 3,83 |
| XLOC_017358 | probable choline kinase 1 | membrane | 24,54 | 121,39 | 11,72 | 13,43 | 13,91 | 13,29 |
| XLOC_028695 | probable aminopyrimidine mitochondrial | mitochondrium | 3,58 | 24,39 | 1,55 | 3,75 | 3,85 | 5,30 |
| XLOC_019766 | D -box ATP-dependent RNA helicase D 17 | nucleic acid processing | 1,13 | 4,93 | 1,06 | 1,21 | 1,08 | 2,38 |
| XLOC_022780 | transcription initiation factor TFIID | nucleic acid processing | 2,31 | 11,83 | 2,85 | 2,59 | 2,00 | 2,24 |
| XLOC_059020 | replication factor A 1 | nucleic acid processing | 0,00 | 7,04 | 0,00 | 7,55 | 1,82 | 0,61 |
| XLOC_020239 | phosphoinositide  phospholipase C 4 | signaling protein | 4,76 | 28,66 | 5,32 | 4,62 | 3,71 | 8,09 |
| XLOC_046056 | transcription factor bHLH47 | signaling protein | 2,22 | 11,96 | 2,65 | 2,44 | 2,64 | 2,25 |
| XLOC_052645 | calcium-binding PBP1 | signaling protein | 6,11 | 42,93 | 1,78 | 2,61 | 2,48 | 3,38 |
| XLOC_055453 | LURP-one-related 12 protein | signaling protein | 2,15 | 10,16 | 4,56 | 2,13 | 2,05 | 2,39 |
| XLOC_024607 | S-type anion channel SLAH2 | transmembrane transport | 7,42 | 34,59 | 14,58 | 13,62 | 17,99 | 19,97 |

**Table S3.** Changes in expression levels (relative to control) of selected transcripts responding to salt stress or shock treatments in *B. maritima* or *B. vulgaris* cv. 'Huzar'. Numbers in cell represent either expression fold ratio for up-regulated genes or reverse of fold ratio for down-regulated ones (denoted with a minus sign). Salt stress – white filling, salt shock – grey filling. ns – not significant difference of gene expression at FDR <0.01.

| gene Id. | annotation | relative expression level | | | |
| --- | --- | --- | --- | --- | --- |
|  |  | MKvsM150 | MKvsM300 | HKvsH150 | HKvsH300 |
| **transcription factor and signaling proteins** | | | | | |
| XLOC_034486 | transcription factor bHLH137 | ns | 4,0 | ns | 4,2 |
| XLOC_018773 | transcription factor WRKY 7 | ns | ns | -3,74 | -5,61 |
| XLOC_046883 | transcription factor WRKY 25 | ns | ns | -5,35 | -3,39 |
| XLOC_042459 | transcription factor WRKY 33 | ns | ns | -4,42 | -3,94 |
| XLOC_056106 | transcription factor WRKY 40 | ns | ns | -15,50 | -8,36 |
| XLOC_030715 | transcription factor WRKY 41 | ns | ns | -4,39 | -3,18 |
| XLOC_015627 | transcription factor WRKY 53 | ns | ns | -4,74 | -4,51 |
| XLOC_042371 | transcription factor WRKY 70 | ns | ns | -4,61 | -5,54 |
| XLOC_027915 | transcription factor NAC 2 | ns | ns | -3,23 | -4,18 |
| XLOC_054938 | transcription factor NAC 29 | ns | ns | -4,12 | ns |
| XLOC_040385 | transcription factor NAC 35 | ns | ns | -2,92 | -2,82 |
| XLOC_056141 | transcription factor MYB44 | ns | ns | -8,07 | -7,49 |
| XLOC_036346 | transcription factor MYB44 | ns | ns | -4,32 | -5,91 |
| XLOC_048035 | SCARECROW protein 13 | ns | ns | -4,50 | -3,72 |
| XLOC_046793 | SCARECROW protein 14 | ns | ns | ns | -3,24 |
| XLOC_003125 | SCARECROW protein 21 | ns | ns | -7,45 | -8,48 |
| XLOC_029550 | receptor kinase THESEUS 1 | ns | ns | -2,94 | -2,31 |
| XLOC_020005 | receptor kinase FERONIA | ns | ns | -3,61 | -3,21 |
| XLOC_020008 | receptor kinase FERONIA | ns | ns | ns | -3,23 |
| XLOC_042625 | receptor kinase FERONIA | ns | ns | -3,49 | -2,77 |
| XLOC_019417 | serine-threonine kinase D6PK | ns | ns | ns | 6,37 |
| XLOC_046765 | probable phosphatase 2C 24; | ns | ns | 7,92 | 7,06 |
| XLOC_021052 | probable phosphatase 2C 72 | ns | ns | -3,52 | -2,89 |
| XLOC_050090 | probable phosphatase 2C 63 | ns | ns | -3,24 | -2,64 |
| XLOC_016763 | probable phosphatase 2C 10 | ns | ns | -6,94 | -3,86 |
| XLOC_046690 | probable phosphatase 2C 25 | ns | ns | -2,76 | -3,26 |
| XLOC_024077 | serine threonine phosphatase PP1 | ns | ns | -4,65 | -3,08 |
| XLOC_013586 | receptor kinase HAIKU2 | ns | ns | -3,97 | -2,24 |
| XLOC_034486 | transcription factor bHLH137 | 37,27 | 54,307 | 44,29 | 89,29 |
| XLOC_029550 | receptor kinase THESEUS 1 | 5,63 | 8,10 | ns | 7,80 |
| XLOC_042625 | receptor kinase FERONIA | 4,23 | 4,27 | 2,89 | 5,43 |
| XLOC_016018 | calcineurin B 4 | 6,06 | 8,16 | ns | ns |
| XLOC_009003 | calcineurin B 10 | ns | 2,63 | ns | ns |
| **osmoprotectant synthesis** | | | | | |
| XLOC_020326 | delta-1-pyrroline-5-carboxylate synthase | ns | 2,95 | ns | ns |
| XLOC_035155 | betaine aldehyde dehydrogenase | ns | 2,33 | ns | ns |
| XLOC_024847 | delta-1-pyrroline-5-carboxylate dehydrogenase | 3,2 | 4,7 | 3,0 | 4,0 |
| XLOC_020326 | delta-1-pyrroline-5-carboxylate synthase | 3,97 | 6,26 | 3,36 | 2,99 |
| XLOC_035155 | betaine aldehyde dehydrogenase | ns | -3,76 | ns | -2,57 |
| XLOC_050339 | sucrose synthase | 3,97 | 4,52 | 3,45 | 6,29 |
| **transmembrane transport** | | | | | |
| XLOC_055026 | aquaporin PIP2-8 | -3,3 | -3,2 | -2,7 | -3,3 |
| XLOC_047607 | aquaporin TIP1-1 | ns | -3,72 | ns | -2,86 |
| XLOC_056008 | aquaporin TIP2-1 | -2,70 | -16,70 | -4,40 | -8,44 |
| XLOC_023789 | probable cyclic nucleotide-gated ion channel 14 | ns | ns | -2,79 | -2,45 |
| XLOC_004852 | probable cyclic nucleotide-gated ion channel 14 | ns | ns | -2,59 | -2,51 |
| XLOC_007139 | potassium channel KAT1 | ns | ns | -3,04 | -2,47 |
| XLOC_054923 | sodium transporter HKT1 | ns | ns | -2,30 | -3,06 |
| XLOC_023798 | probable voltage-gated potassium channel | ns | 2,23 | ns | ns |
| XLOC_042660 | lipid transfer protein | 18,6 | 41,6 | 13,9 | 15,6 |
| XLOC_020419 | non-specific lipid-transfer protein | 6,4 | 8,3 | 5,2 | 9,4 |
| XLOC_020420 | non-specific lipid-transfer protein | ns | ns | 3,5 | 3,4 |
| XLOC_050739 | sugar transporter 14 | -2,81 | ns | -3,18 | -3,18 |
| XLOC_034363 | bidirectional sugar transporter SWEET14 | ns | -2,46 | -2,35 | ns |
| XLOC_047800 | sugar transporter 1 | ns | ns | -3,22 | -2,92 |
| XLOC_001583 | bidirectional sugar transporter SWEET2 | ns | ns | ns | -3,26 |
| XLOC_034373 | bidirectional sugar transporter SWEET12 | ns | ns | ns | -2,95 |
| XLOC_026206 | NRT1 PTR family protein | 2,6 | 2,8 | 2,7 | 2,2 |
| XLOC_034861 | NRT1 PTR family protein | ns | ns | 2,5 | 2,8 |
| XLOC_038928 | NRT1 PTR family protein | ns | 2,5 | 2,0 | 3,1 |
| XLOC_020904 | NRT1 PTR family protein | ns | ns | ns | 3,8 |
| XLOC_020901 | NRT1 PTR family protein | ns | ns | -2,61 | ns |
| XLOC_055026 | aquaporin PIP2-8 | 5,00 | 7,21 | 3,89 | 5,95 |
| XLOC_019782 | aquaporin PIP1-4 | 4,68 | ns | 3,57 | 5,97 |
| XLOC_047607 | aquaporin TIP1-1 | 5,648 | 6,96 | 7,34 | 10,38 |
| XLOC_056008 | aquaporin TIP2-1 | 18,16 | 16,77 | 17,71 | 27,69 |
| XLOC_053207 | aquaporin PIP2-1 | 2,32 | 4,23 | ns | 4,11 |
| XLOC_054741 | X intrinsic protein | ns | 6,45 | ns | ns |
| XLOC_037202 | aquaporin nip1-1 | ns | ns | -2,89 | ns |
| XLOC_055661 | aquaporin NIP6-1 | ns | ns | ns | -2,74 |
| XLOC_051856 | two-pore potassium channel 3 | 4,69 | 6,25 | ns | ns |
| XLOC_020335 | lipid-transfer protein DIR1 | ns | -10,97 | ns | ns |
| XLOC_024607 | S-type anion channel SLAH2 | 12,20 | 18,28 | 8,42 | 15,37 |
| XLOC_023327 | chloride channel CLC-b | 4,71 | 7,61 | ns | 2,6 |
| XLOC_034418 | chloride channel CLC-b | 3,63 | 2,94 | ns | ns |
| XLOC_012252 | lipid transfer protein | ns | -4,79 | ns | ns |
| XLOC_050060 | non-specific lipid-transfer protein | ns | ns | ns | -3,71 |
| XLOC_020422 | non-specific lipid-transfer protein | ns | -11,67 | -2,20 | -4,06 |
| XLOC_020420 | non-specific lipid-transfer protein | -5,71 | -12,12 | -11,79 | -15,80 |
| XLOC_001583 | bidirectional sugar transporter SWEET2 | 3,92 | 4,73 | ns | 3,74 |
| XLOC_034363 | bidirectional sugar transporter SWEET14 | -2,52 | -2,09 | -3,24 | -3,58 |
| XLOC_011343 | sugar transporter ERD6 | 2,99 | 3,46 | ns | 2,32 |
| XLOC_036699 | sugar transporter ERD6 | 3,08 | 2,57 | ns | 2,17 |
| XLOC_002079 | sugar transporter ERD6 | -2,58 | -2,69 | -2,68 | -4,43 |
| XLOC_041843 | sucrose transporter SUC3 | 2,81 | 3,39 | ns | ns |
| XLOC_034863 | NRT1 PTR family protein | -3,32 | -3,17 | ns | ns |
| XLOC_047065 | NRT1 PTR family protein | -3,02 | -4,10 | ns | ns |
| XLOC_034861 | NRT1 PTR family protein | 3,14 | 4,51 | 4,72 | 3,87 |
| XLOC_020904 | NRT1 PTR family protein | -4,96 | -10,64 | ns | ns |
| XLOC_041874 | NRT1 PTR family protein | ns | -10,27 | ns | ns |
| XLOC_020906 | NRT1 PTR family protein | ns | -3,32 | ns | ns |
| **cell wall plasticity** | | | | | |
| XLOC_054945 | expansin A2 | ns | ns | -13,44 | -6,25 |
| XLOC_054944 | expansin A2 | ns | ns | -4,63 | -3,74 |
| XLOC_023390 | expansin A8 | ns | ns | ns | 2,65 |
| XLOC_031677 | expansin B1 | ns | ns | ns | 7,36 |
| XLOC_023534 | expansin A10 | 2,7 | 3,3 | 4,0 | 9,1 |
| XLOC_016696 | classical arabinogalactan 5 | ns | ns | -2,91 | -2,89 |
| XLOC_029803 | fasciclin-like arabinogalactan 1 | ns | -6,93 | ns | -2,76 |
| XLOC_019290 | fasciclin-like arabinogalactan 8 | -3,40 | ns | -4,30 | -3,50 |
| XLOC_046661 | fasciclin-like arabinogalactan 17 | ns | -2,78 | ns | -2,13 |
| XLOC_032732 | fasciclin-like arabinogalactan 17 | ns | ns | -13,97 | -6,37 |
| XLOC_023061 | xyloglucan endotransglycosylase hydrolase | ns | ns | -24,88 | -19,95 |
| XLOC_016782 | xyloglucan endotransglycosylase hydrolase | ns | ns | -24,26 | -9,06 |
| XLOC_026567 | xyloglucan endotransglucosylase hydrolase 2 | ns | ns | -3,55 | -2,64 |
| XLOC_026612 | xyloglucan endotransglucosylase hydrolase 2 | ns | ns | ns | -4,53 |
| XLOC_013374 | xyloglucan endotransglucosylase hydrolase 9 | ns | ns | -3,93 | -4,41 |
| XLOC_032493 | probable xyloglucan endotransglucosylase hydrolase 30 | ns | ns | -2,42 | -2,72 |
| XLOC_023975 | cellulose synthase D3 | ns | ns | -2,79 | -2,39 |
| XLOC_034939 | cellulose synthase | ns | ns | -3,41 | -2,78 |
| XLOC_018851 | cinnamoyl- reductase 1 | 3,7 | 4,1 | 6,9 | 9,3 |
| XLOC_027566 | cinnamoyl- reductase 1 | ns | ns | -2,25 | -2,73 |
| XLOC_016733 | EXORDIUM protein | -3,67 | -6,2 | -32,9 | -18,9 |
| XLOC_024815 | EXORDIUM 3 protein | ns | -3,37 | ns | -2,48 |
| XLOC_016422 | EXORDIUM protein | ns | ns | -34,34 | -14,38 |
| XLOC_054770 | EXORDIUM 2 protein | ns | ns | -3,58 | -6,38 |
| XLOC_055175 | EXORDIUM protein | ns | - | -7,72 | -8,08 |
| XLOC_023895 | COBRA 7 protein | ns | ns | -12,43 | -6,90 |
| XLOC_023390 | expansin A8 | 10,39 | 20,44 | 14,06 | 27,05 |
| XLOC_023534 | expansin A10 | 20,22 | 35,29 | 27,87 | 62,29 |
| XLOC_041792 | expansin A10 | 4,27 | 6,54 | 4,98 | 10,72 |
| XLOC_023534 | expansin A10 | 20,22 | 35,29 | 27,87 | 62,29 |
| XLOC_054945 | expansin | 6,68 | 7,55 | 11,10 | 14,31 |
| XLOC_031677 | expansin | -2,96 | -3,85 | ns | -8,03 |
| XLOC_054944 | expansin | ns | ns | 23,48 | 47,05 |
| XLOC_054944 | expansin | ns | ns | ns | 47,05 |
| XLOC_043390 | fasciclin-like arabinogalactan 11 | ns | -4,76 | ns | ns |
| XLOC_029803 | fasciclin-like arabinogalactan 1 | 2,89 | 2,74 | 6,20 | 9,48 |
| XLOC_016696 | classical arabinogalactan 5 | 26,63 | 37,66 | 19,68 | 25,96 |
| XLOC_019290 | fasciclin-like arabinogalactan 8 | 3,88 | 3,24 | 4,76 | 5,22 |
| XLOC_053760 | fasciclin-like arabinogalactan 9 | 88,85 | ns | ns | ns |
| XLOC_025123 | arabinogalactan peptide 13 | 6,93 | 7,13 | 3,17 | 6,29 |
| XLOC_032732 | fasciclin-like arabinogalactan 17 | 4,75 | 3,79 | ns | ns |
| XLOC_046661 | fasciclin-like arabinogalactan 17 | 2,97 | 3,28 | 3,55 | 5,045 |
| XLOC_036455 | lysine-rich arabinogalactan 18 | 35,17 | 51,64 | 10,16 | 14,66 |
| XLOC_026612 | xyloglucan endotransglucosylase hydrolase | 9,90 | 14,01 | 5,89 | 11,06 |
| XLOC_034939 | cellulose synthase | 4,79 | 6,37 | 4,29 | 6,83 |
| XLOC_008725 | cellulose synthase | 3,02 | 3,32 | 3,14 | 4,53 |
| XLOC_053223 | cellulose synthase | 2,16 | 2,72 | 2,04 | 2,74 |
| XLOC_024980 | cellulose synthase | -4,53 | -9,54 | -3,83 | -4,78 |
| XLOC_023975 | cellulose synthase | 3,85 | 5,26 | 3,91 | 5,94 |
| XLOC_023561 | cellulose synthase | 3,99 | 5,13 | 3,72 | 5,79 |
| XLOC_017285 | cellulose synthase | 2,48 | 2,91 | 3,01 | 3,54 |
| XLOC_018851 | cinnamoyl- reductase 1 | -11,04 | -88,27 | -5,44 | -24,83 |
| XLOC_054770 | EXORDIUM 2 protein | -4,84 | -6,49 | -4,43 | -4,26 |
| XLOC_016733 | EXORDIUM protein | 74,72 | 88,74 | ns | ns |
| XLOC_024815 | EXORDIUM 3 protein | ns | -2,92 | ns | ns |
| XLOC_023895 | COBRA 7 protein | 13,52 | 17,57 | 7,11 | 13,35 |
| **pathogen response** | | | | | |
| XLOC_023035 | hypersensitive-induced response 1 protein | ns | 4,6 | ns | 4,3 |
| XLOC_007448 | chitinase | -13,76 | ns | ns | ns |
| XLOC_016322 | endochitinase | ns | ns | -2,58 | -4,75 |
| XLOC_031638 | ribosome-inactivating protein | nd | -12,36 | nd | nd |
| XLOC_008632 | ribosome-inactivating protein | nd | nd | nd | -4,26 |
| XLOC_048458 | ribosome-inactivating protein | nd | nd | -10,85 | -24,04 |
| XLOC_015324 | ribosome-inactivating protein | nd | nd | nd | -4,17 |
| XLOC_046291 | osmotin | ns | 5,4 | 3,1 | 4,4 |
| XLOC_022648 | pore-forming toxin Hfr-2 | nd | nd | 3,7 | 3,4 |
| XLOC_022659 | pore-forming toxin Hfr-2 | nd | nd | nd | 3,7 |
| XLOC_049136 | pore-forming toxin Hfr-2 | -4,41 | -7,4 | nd | nd |
| XLOC_034905 | defensin J1-2 | ns | 3,2 | ns | 2,2 |
| XLOC_019626 | defensin AX1 | ns | ns | -3,09 | -4,18 |
| XLOC_019649 | defensin AX1 | ns | ns | -2,17 | -3,92 |
| XLOC_030805 | thaumatin | 3,85 | 6,20 | ns | ns |
| XLOC_014663 | disease resistance protein RPP13 | ns | ns | 9,74 | 7,67 |
| XLOC_055120 | snakin-2 | ns | -3,07 | ns | -3,06 |
| XLOC_023035 | hypersensitive-induced response protein 1 | 3,89 | 4,66 | 2,62 | 7,78 |
| XLOC_034094 | chitinase 1 | 2,63 | 2,51 | 3,03 | 4,25 |
| XLOC_037700 | chitotriosidase 1 | 11,69 | 4,69 | ns | ns |
| XLOC_049921 | endochitinase EP3 | -4,34 | ns | ns | ns |
| XLOC_016322 | endochitinase | ns | ns | ns | -3,04 |
| XLOC_024222 | acidic endochitinase | ns | ns | ns | -6,14 |
| XLOC_031638 | ribosome-inactivating protein | -11,85 | -13,83 | nd | nd |
| XLOC_015324 | ribosome-inactivating protein | -2,77 | -3,26 | -4,55 | -6,97 |
| XLOC_008632 | ribosome-inactivating protein | nd | nd | -3,95 | -4,33 |
| XLOC_051725 | ribosome-inactivating lychnin protein | -7,83 | nd | nd | nd |
| XLOC_053015 | ribosome-inactivating lychnin protein | nd | -3,53 | nd | nd |
| XLOC_003677 | type 2 ribosome-inactivating precursor protein | -6,90 | -7,42 | -4,36 | -6,65 |
| XLOC_022659 | pore-forming toxin Hfr-2 | -9,74 | -17,97 | -2,77 | -4,85 |
| XLOC_049136 | pore-forming toxin Hfr-2 | -7,57 | -8,86 | -5,53 | -8,62 |
| XLOC_022648 | pore-forming toxin Hfr-2 | -2,89 | nd | nd | -2,33 |
| XLOC_040779 | pore-forming toxin Hfr-2 | -2,32 | -3,17 | nd | nd |
| XLOC_034910 | defensin | 4,58 | 4,44 | 4,66 | 3,77 |
| XLOC_034905 | defensin J1-2 | ns | ns | ns | 3,21 |
| \XLOC_034904 | defensin J1-2 | ns | -4,49 | -3,74 | ns |
| XLOC_034909 | defensin | ns | -4,15 | ns | ns |
| XLOC_011303 | remorin | 3,62 | 4,19 | 3,40 | 3,91 |
| XLOC_026017 | remorin | -3,65 | -2,53 | ns | -6,99 |
| XLOC_006157 | remorin | 2,47 | ns | ns | 3,75 |
| XLOC_030806 | thaumatin | 3,04 | ns | -13,65 | ns |
| XLOC_030805 | thaumatin | ns | ns | -4,57 | -12,93 |
| XLOC_055120 | snakin-2 | 6,07 | 10,18 | ns | ns |
| **chloroplast functions** | | | | | |
| XLOC_021719 | ribulose bisphosphate carboxylase, small subunit, chloroplastic | -4,8 | -4,2 | -3,5 | -4,8 |
| XLOC_004905 | chlorophyll a-b binding protein, chloroplastic | -9,90 | -6,8 | -4,9 | -11,2 |
| XLOC_004937 | chlorophyll a-b binding protein, chloroplastic | ns | -4,46 | -4,46 | -5,84 |
| XLOC_019946 | ribulose bisphosphate carboxylase small subunit, chloroplastic | ns | -3,76 | ns | ns |
| XLOC_030371 | chlorophyll a-b binding protein, chloroplastic | -5,39 | -15,67 | -3,49 | -7,55 |
| XLOC_004905 | chlorophyll a-b binding protein, chloroplastic | -4,64 | ns | ns | ns |
| XLOC_023442 | chlorophyll a-b binding protein, chloroplastic | ns | -4,98 | ns | -3,12 |
| XLOC_004937 | chlorophyll a-b binding protein, chloroplastic | -3,81 | -6,01 | ns | -2,43 |
| XLOC_034698 | chlorophyll a-b binding protein, chloroplastic | ns | -4,58 | ns | ns |
| XLOC_015655 | chlorophyll a-b binding protein, chloroplastic | ns | -3,72 | ns | ns |
| XLOC_004936 | chlorophyll a-b binding protein, chloroplastic | ns | -3,15 | ns | -3,38 |
| XLOC_007571 | chlorophyll a-b binding protein, chloroplastic | -3,43 | -7,67 | -2,51 | -4,01 |
| XLOC_001112 | chlorophyll a-b binding protein, chloroplastic | -3,38 | -5,53 | ns | -2,82 |
| XLOC_042208 | chlorophyll a-b binding protein, chloroplastic | ns | -3,55 | ns | ns |
| XLOC_029711 | chlorophyll a-b binding protein, chloroplastic | ns | ns | -2,66 | ns |
| XLOC_055997 | photosystem I reaction center subunit chloroplastic | ns | -3,13 | ns | ns |
| XLOC_026622 | photosystem I reaction center subunit chloroplastic | ns | -2,99 | ns | ns |
| XLOC_046953 | photosystem I reaction center subunit chloroplastic | ns | -2,78 | ns | ns |
| XLOC_036934 | photosystem I reaction center subunit chloroplastic | ns | -2,69 | ns | ns |
| XLOC_040834 | photosystem II core complex s, chloroplastic | -2,71 | -4,48 | ns | -2,09 |
| XLOC_035543 | chloroplast photosystem II protein | ns | -3,03 | ns | ns |
| **secondary metabolism** | | | | | |
| XLOC_056068 | cytochrome P450 76AD1 | -11,5 | ns | ns | -5,1 |
| XLOC_021069 | cytochrome P450 CYP72A219 | ns | -11,49 | ns | ns |
| XLOC_030784 | cytochrome P450 86A22 | ns | -3,28 | -2,01 | -2,77 |
| XLOC_003448 | cytochrome P450 710A1 | ns | ns | -4,73 | -3,41 |
| XLOC_006064 | non-functional NADPH-dependent codeinone reductase 2 | 7,8 | 8,5 | 4,1 | 5,2 |
| XLOC_003448 | cytochrome P450 710A1 | 9,02 | 12,35 | 7,85 | 12,71 |
| XLOC_020016 | cytochrome P450 85A | 5,83 | 8,90 | 6,18 | 10,55 |
| XLOC_016877 | cytochrome P450 83B1 | 4,93 | 2,68 | 4,30 | 4,39 |
| XLOC_047048 | cytochrome P450 71A1 | 3,26 | 4,78 | 2,20 | 2,64 |
| XLOC_011234 | cytochrome P450 81F3 | 3,66 | 3,79 | 3,42 | 5,14 |
| XLOC_000430 | cytochrome P450 CYP72A219 | 2,72 | 3,14 | ns | 2,38 |
| XLOC_050350 | cytochrome P450 71D10 | -30,83 | ns | -9,38 | -35,48 |
| XLOC_055916 | cytochrome P450 | -2,73 | -4,39 | -3,42 | ns |
| XLOC_046047 | cytochrome P450 71A1 | ns | -3,95 | -3,39 | ns |
| XLOC_021069 | cytochrome P450 CYP72A219 | ns | -3,53 | ns | ns |
| XLOC_047116 | cytochrome P450 71A26 | ns | -2,88 | ns | ns |
| XLOC_030784 | cytochrome P450 86A22 | ns | ns | 3,98 | 6,94 |
| XLOC_041787 | cytochrome P450 704B1 | ns | ns | ns | -3,59 |
| XLOC_036912 | cytochrome P450 chloroplastic | ns | ns | -2,61 | -2,81 |
| XLOC_006064 | non-functional NADPH-dependent codeinone reductase | -2,45 | -5,69 | ns | -5,07 |
| **protein processing** | | | | | |
| XLOC_050471 | E3 ubiquitin ligase RHY1A | ns | ns | -3,64 | -4,04 |
| XLOC_052448 | E3 ubiquitin ligase MIEL1 | ns | ns | -3,72 | ns |
| XLOC_024057 | E3 ubiquitin ligase XBAT35 | ns | ns | -2,57 | ns |
| XLOC_012437 | E3 ubiquitin ligase ATL6 | ns | ns | -6,51 | -5,74 |
| XLOC_026093 | E3 ubiquitin ligase MARCH2 | ns | ns | -2,39 | -2,66 |
| XLOC_036777 | ubiquitin-conjugating enzyme  E2-17 kDa | ns | ns | -5,44 | -3,52 |
| XLOC_013200 | E3 ubiquitin ligase KEG | -2,79 | -2,60 | ns | ns |
| XLOC_044438 | E3 ubiquitin ligase BAH1 | ns | 6,41 | ns | ns |
| XLOC_008276 | E3 ubiquitin ligase RIE1 | ns | 3,33 | ns | ns |
| XLOC_027341 | ubiquitin-conjugating enzyme E2 | ns | ns | ns | 3,61 |
| XLOC_015624 | polyubiquitin | ns | 3,41 | ns | ns |
| **other genes** | | | | | |
| XLOC_056368 | phloem filament PP1 | 7,4 | 26,1 | 6,6 | 16,8 |
| XLOC_001368 | ureide permease | ns | 14,0 | 12,8 | 17,5 |
| XLOC_024568 | bark storage A protein | ns | ns | 87,7 | 350,4 |
| XLOC_024569 | bark storage A protein | ns | ns | 3,3 | 6,0 |
| XLOC_020807 | bark storage A protein | ns | -5,3 | ns | -3,8 |
| XLOC_024108 | bark storage A protein | ns | -4,08 | ns | ns |
| XLOC_024568 | bark storage A protein | -11,25 | -99,15 | ns | -8,20 |
| XLOC_024108 | bark storage A protein | -8,48 | ns | ns | -15,57 |
| XLOC_020807 | bark storage A protein | -3,88 | -26,09 | ns | -3,61 |
| XLOC_020806 | bark storage A protein | ns | -8,48 | ns | ns |

**Table S4.** Sequence of PCR primers used to validate RNASeq results.

| gene Id. | |  | primer sequence (5’->3’) | primer length [bp] | Tm [ºC] | GC [%] | PCR product length [bp] |
| --- | --- | --- | --- | --- | --- | --- | --- |
| XLOC_006064 | codeinone reductase | F | GTTGCCCAGGTTGCTTTG | 18 | 60,2 | 55,6 | 93 |
|  |  | R | TCCGAGGTTCTGTTTCATCC | 20 | 60,0 | 50,0 |  |
| XLOC_015624 | poliubiqutin | F | CCTTCACCTTGTCCTTCGTC | 20 | 59,7 | 55,0 | 90 |
|  |  | R | CCCACACATAACACCCAGAG | 20 | 58,9 | 55,0 |  |
| XLOC_030667 | amine oxidase | F | CGGGTTGAAAAGGTTGATGT | 20 | 59,8 | 45,0 | 158 |
|  |  | R | ATGGGTAAGCAAGCCAAGTC | 20 | 59,2 | 50,0 |  |
| XLOC_024847 | aldehyde dehydrogenase | F | GGAGTTCAGTAAATGGGACGA | 21 | 59,0 | 47,6 | 126 |
|  |  | R | GCAACGCTTCAGGTAGATGG | 20 | 60,8 | 55,0 |  |
| XLOC_041948 | β-glucosidase | F | TTGAAGCAAGCAAAGGAAGG | 20 | 60,5 | 45,0 | 106 |
|  |  | R | GCGATGGAAAAACGGTAAGA | 20 | 60,1 | 45,0 |  |
| XLOC_035184 | α,α-trehalose-phosphate synthase | F | GTCAAAACTCCAAGCCCTGA | 20 | 60,2 | 50,0 | 89 |
|  |  | R | CCCCTGCCACTAACAATGAA | 20 | 60,9 | 50,0 |  |
| XLOC_035693 | β-D-xylosidase | F | TCGGATTGTGATTCTGTTGG | 20 | 59,5 | 45,0 | 149 |
|  |  | R | CCCTTCTTTATGGCGTCTTCT | 21 | 59,7 | 47,6 |  |
| XLOC_034939 | cellulose synthase | F | GCATCTTTACCCCTTCCTCA | 20 | 59,1 | 50,0 | 171 |
|  |  | R | TCACAATCCAACCCACAAAC | 20 | 59,2 | 45,0 |  |
| XLOC_042404 | serine/threonine-protein kinase WNK5 | F | GATTGGTGATTTGGGATTGG | 20 | 60,0 | 45,0 | 189 |
|  |  | R | TTTGAGCATTCGCTGTAAGG | 20 | 59,1 | 45,0 |  |
| XLOC_004937 | chlorophyll a/b binding protein | F | ATGGGACACTGCTGGACTCT | 20 | 59,7 | 55,0 | 129 |
|  |  | R | TTGCGGGCTAACAACTCTG | 19 | 60,0 | 52,6 |  |

**Table S5.** RT-qPCR confirmation of selected transcripts identified by RNA-sequencing. Numbers without brackets show relative expression levels issuing from RT-qPCR experiment, highlighted numbers with brackets show RNASeq results. Negative values indicate that expression was x times smaller in the second of the listed treatments than in the first one. (ns) denotes not significant change of expression. Otherwise, the values represent significant change in the gene expression level at p < 0.01.

| gene Id. | SALT SHOCK | | | | | |
| --- | --- | --- | --- | --- | --- | --- |
|  | relative expression level - RT-qPCR **(RNASeq)** | | | | | |
|  | MK vs M150 | MK vs M300 | M150 vs M300 | HK vs H150 | HK vs H300 | H150 vs H300 |
| codeinone reductase  XLOC_006064 | -1,10 **(-2,45)** | -3,64 **(-5,69)** | -3,30 **(ns)** | -2,51 **(-3,01)** | -2,73 **(-5,06)** | -3,31 **(ns)** |
| poliubiqutin  XLOC_015624 | 3,17 **(ns)** | 5,31 **(3,41)** | 1,67 **(ns)** | 1,61  **(ns)** | 1,78 **(ns)** | -1,09 **(ns)** |
| amine oxidase  XLOC_030667 | 1,38  **(ns)** | -1,56 **(ns)** | -1,85 **(ns)** | 1,03 **(ns)** | -1,62 **(-2,59)** | -1,67 **(ns)** |
| aldehyde dehydrogenase  XLOC_024847 | 1,46 **(ns)** | 1,49 **(ns)** | 1,02 **(ns)** | -1,52  **(ns)** | - 1,78 **(ns)** | -1,16 **(ns)** |
| β-glucosidase  XLOC_041948 | -5,29 **(-23,27)** | -44,45 **(ns)** | -8,41  **(ns)** | -1,74 **(-4,99)** | -14,66 **(-27,53)** | -8,45  **(ns)** |
| α,α-trehalose-phosphate synthase  XLOC_035184 | 8,35 **(11,72)** | 22,89  **(15,57)** | 1,44  **(ns)** | 7,80  **(4,57)** | 19,02 **(10,80)** | 2,44 **(2,36)** |
| β-D-xylosidase  XLOC_035693 | 3,77 **(6,67)** | 16,71 **(13,31)** | 4,44 **(ns)** | 3,95 **(4,64)** | 9,54 **(10,38)** | 2,42 **(ns)** |
| cellulose synthase  XLOC_034939 | 2,66 **(4,79)** | 9,97 **(6,36)** | 3,75 **(ns)** | 5,59 **(4,28)** | 7,27 **(6,83)** | 1,30 **(ns)** |
| serine/threonine-protein kinase WNK5  XLOC_042404 | -2,18 **(-5,00)** | -10,31 **(-8,17)** | -4,72 **(ns)** | -2,72 **(-3,62)** | -3,87 **(-7,81)** | -1,42 **(-2,15)** |
| chlorophyll a/b binding protein  XLOC_004937 | 1,25 **(-3,81)** | -2,19 **(-6,00)** | -2,73 **(ns)** | 1,17 **(ns)** | -1,69 **(-2,42)** | -1,98 **(-2,03)** |
|  | **SALT STRESS** | | | | | |
| codeinone reductase  XLOC_006064 | 3,46 **(7,80)** | 2,37 **(8,50)** | -1,46 **(ns)** | 5,06 **(4,15)** | 8,74 **(5,29)** | 1,73 **(ns)** |
| poliubiqutin  XLOC_015624 | 3,27 **(3,92)** | 5,24 **(6,07)** | 1,60 **(ns)** | 4,92 **(4,66)** | 6,66 **(6,03)** | 1,35 **(ns)** |
| amine oxidase  XLOC_030667 | 13,54 **(4,45)** | 9,91 **(4,48)** | -1,37  **(ns)** | 1,78  **(ns)** | 1,04  **(ns)** | -1,70  **(ns)** |
| aldehyde dehydrogenase  XLOC_024847 | 2,39 **(3,26)** | 3,09 **(4,74)** | 1,29 **(ns)** | 2,27 **(3,02)** | 3,58 **(4,00)** | -1,57 **(ns)** |
| β-glucosidase  XLOC_041948 | -30,21 **(-13,53)** | -27,02 **(-17,00)** | 1,12 **(ns)** | 3,84 **(2,33)** | 12,86 **(4,08)** | 3,35 **(ns)** |
| α,α-trehalose-phosphate synthase  XLOC_035184 | -1,30 **(ns)** | 1,21 **(ns)** | 1,57 **(ns)** | -9,30 **(-4,10)** | -4,67 **(-3,98)** | 1,99 **(ns)** |
| β-D-xylosidase  XLOC_035693 | -2,18 **(-3,77)** | -1,43 **(-4,01)** | 1,52 **(ns)** | -20,87 **(-7,36)** | -8,97 **(-10,12)** | 2,32  **(ns)** |
| cellulose synthase  XLOC_034939 | -1,71 **(ns)** | -1,52 **(ns)** | 1,12 **(ns)** | -3,06 **(-3,40)** | -1,69 **(-2,77)** | 1,80 **(ns)** |
| serine/threonine-protein kinase WNK5  XLOC_042404 | 1,04 **(ns)** | -1,46  **(ns)** | -1,50 **(ns)** | 1,13 **(ns)** | 1,59 **(ns)** | 1,40 **(ns)** |
| chlorophyll a/b binding protein  XLOC_004937 | -2,96 **(ns)** | -5,12 **(-4,46)** | -1,73 **(ns)** | -5,14 **(-4,45)** | -5,95 **(-5,84)** | -1,16 **(ns)** |
