# Supplementary material for: Salt stress vs. salt shock - the case of sugar beet and its halophytic ancestor
Source: BMC Plant Biol. 2019 Feb 6;19:57. doi: 10.1186/s12870-019-1661-x (PMC6364445; doi:10.1186/s12870-019-1661-x)
Supplement: Supplementary file 2 — Supplementary Methods. (DOC 97 kb) [file 12870_2019_1661_MOESM2_ESM.doc]

**Salt stress vs. salt shock - the case of sugar beet and its halophytic ancestor**

Monika Skorupa^1*^, Marcin Gołębiewski^1,2*^, Katarzyna Kurnik^2^, Janusz Niedojadło^3^, Jacek Kęsy^2^, Krzysztof Klamkowski^4^, Katarzyna Wójcik^4^, Waldemar Treder^4^, Andrzej Tretyn^1,2^, Jarosław Tyburski^1,2*#^

^1^ Centre for Modern Interdisciplinary Technologies, Nicolaus Copernicus University, Toruń, Poland; ^2^ Chair of Plant Physiology and Biotechnology, Faculty of Biology and Environment Protection, Nicolaus Copernicus University, Toruń, Poland; ^3^Department of Cell Biology, Faculty of Biology and Environment Protection, Nicolaus Copernicus University, Toruń, Poland; ^4^ Research Institute of Horticulture, Skierniewice, Poland

*** -** these authors contributed equally to this work

# - corresponding author, addresses:

Author for correspondence:

*Jarosław Tyburski*

*Tel: +48 56 611 47 73*

*Email: tybr@biol.umk.pl*

**RNA isolation, sequencing library preparation, their quantification, multiplexing and sequencing**

Total RNA was isolated from leaf samples using TriPure reagent (Roche) and subjected to DNase treatment with DNase I (Thermo Scientific). RNA quantity and quality was determined using Qubit fluorometer (Life Technologies) and by agarose gel electrophoresis. RNA integrity was checked using RNA 6000 Nano Assay Kit and Agilent 2100 Bioanalyzer (Agilent Technologies). Sequencing libraries were prepared from 1 µg of total RNA using TruSeq Stranded Total RNA Sample Preparation Kits with Ribo-Zero Plant (Illumina). Following synthesis, libraries were subjected to quantitation by qPCR using KAPPA Illumina library quantification kit (KappaBiosystems) and quality control by means of Bioanalyzer DNA HS assay kit and Agilent 2100 Bioanalyzer (Agilent Technologies). Individual libraries were pooled using the same amount of each library and the quality of the final library pool was assessed with Bioanalyzer, as described above. Finally, the libraries were sequenced using MiSeq Reagent v3 Kit on MiSeq sequencer (Illumina). The bioinformatic analysis of the RNASeq data was performed as described below.

**Processing and analysis of RNASeq data**

**(1) Short reads quality trimming.** The quality trimming was performed with prinseq lite . Sequence stretches with average quality <30 over a window of 20 nt were removed, and the reads were cut immediately before the first incidence of a degenerated base. Furthermore, the trimmed reads shorter than 50 nt were removed. **(2) Mapping the short reads to the reference genome and transcriptome and identification of differentially expressed genes.** Tuxedo suite programs were used to perform mapping and DEGs identification, 24 cores were used throughout the computations and the RAM limit was set to 96 GB. Tophat2 aligner v2.0.10 9 with Bowtie2 engine v2.1.0 was used to map the trimmed reads to the reference (RefBeet v.1.1downloaded from http://bvseq.molgen.mpg.de/Genome/Download/RefBeet-1.1) genome and transcriptome, which enabled proper dealing with the problem of non-uniquely mapped reads and allowed identification of reads spanning splice junctions. One mismatch per read was allowed to account for unavoidable sequencing errors. Aligned reads were then assembled with Cufflinks v2.1.1 to identify different variants of transcripts. All the assemblies were merged with Cuffmerge and the merged set of transcripts served as a reference for transcript quantitation with Cuffquant. The last step was identification of DEGs with Cuffdiff v2.1.1 . Contrasts were defined using the sample sheet and contrasts file (i.e. --use-sample-sheet and -C options were used). Significance threshold of 0.01 was assumed. All the programs were run with default settings. Counts of 8 specific transcript categories were obtained with custom-tailored Perl scripts. **(3) GO enrichment analysis.** topGO v.2.20.0 package within R v.3.2.2 was used. The data obtained from cuffdiff and RefBeet v.1.1 annotations were used to generate lists of differentially expressed genes with GO terms, custom Perl script was used for this purpose. Weight01 algorithm was used in conjunction with Fisher's exact test, only categories with at least 10 DEGs were analyzed. **(4) Analysis of bHLH binding sites (E-boxes) incidence.** Promoter regions of all annotated genes (i.e. 1500 bp upstream of transcription start sites) were extracted from genome fasta file were extracted and presence of a canonical E-box sequence (CANNTG) was determined with a Perl script. Then lists of all genes and DEGs were prepared with R based on genome reference and cuffdiff analysis, respectively, and a list of bHLH binding site-bearing promoter regions was obtained. Counts were organized in a 2 x 2 contingency table and two-tailed Fisher’s exact test was performed using fisher.test in R. **(5) Analysis of bHLH137 (Bv_111360_mzap) promoter region.** 1500 bp upstream of the gene's transcription start site were analyzed with PlantPAN 2.0 at default parameters values. **(6) Construction of bHLH137 phylogenetic tree.** Putative *Bv*bHLH137 homologs were identified in NCBI GenPept database using BLASTp (word size 2, expect threshold 0.0001, otherwise default parameters), downloaded and aligned using Clustal Omega. The tree was calculated with Phyml 20131022 with the default set of parameters.

**Experimental validation of differential expression data by RT-qPCR**

In order to validate differential gene expression, RT qPCR analysis was performed. Total RNA was isolated from the same leaf samples a using TriPure reagent (Roche). The amount of 5 µg of total RNA preparations was subjected to DNase treatment with DNase I (Thermo Scientific). Following the Dnase I digestion, the first-strand cDNA was synthesized from 1 µg of total RNA using random hexamers and Transcriptor High Fidelity cDNA Synthesis Kit (Roche), following the manufacturer’s instructions. Gene specific primers were designed using Primer3Plus software. To ensure correctness of the quantification, the expression was normalized to the 18S rRNA reference gene. The stability of 18S rRNA expression levels among the samples was assessed using *BestKeeper* Software, as described by Skorupa-Kłaput et al. . In order to determine the PCR efficiencies, standard curves for both target and control genes were obtained using a five-fold series of cDNA dilutions as a template. RT qPCR was performed in three biological replicates, each of which consisted of three technical replicates, using FastStart SYBR Green Master mix following the standard PCR program suggested by the manufacturer (Roche). The relative level of gene expression was calculated according to the Pfaffl method using the REST v2.0.13 2009 software. Blank controls without cDNA template were included in every experimental set up. List of PCR primers used or experimental validation is provided in Table S4.

To validate the results of transcriptome profiling performed we assessed the expression of candidate DEGs with RT qPCR and compared the results with gene expression data from RNA-Seq (Table S5). We selected ten genes for the analysis: codeinone reductase (XLOC_006064), poliubiqutin (XLOC_015624), amine oxidase (XLOC_030667), aldehyde dehydrogenase (XLOC_024847), β-glucosidase (XLOC_041948), α,α-trehalose-phosphate synthase (XLOC_035184), β-D-xylosidase (XLOC_035693), cellulose synthase (XLOC_034939), serine/threonine-protein kinase WNK5 (XLOC_042404), chlorophyll a/b binding protein (XLOC_004937). The results of qPCR analysis showed agreement of gene expression profiles with the predictions derived from the RNA-Seq approach (Table S5).

**Fluorescence in situ hybridisation (FISH) of polyA RNA and 25S rRNA and cell volume measurements.**

The leaves were fixed in 4% formaldehyde (Polysciences) diluted in PIPES (Sigma-Aldrich) for 1 h, under vacuum and washed three times for 10 min in PBS. Fixed leaves were disrupted in order to obtain a fraction of singular cells by gentle homogenisation in Potter homogeniser, according to modified protocol of Tirichine et al. . The suspension of mesophyll cells was spread on biobond coated slides placed on dry ice and air-dried. FISH detection of polyA RNA and rRNA was accomplished according to the Dełeńko et al. (2015) with modifications. For the analysis of 25S rRNA the oligo probe 5′Cy3-TTC GCA GTT TCA CAG TCT GAA TTA GTT CAT-3′was used at a concentration of 50 pmol/ml, and hybridization was performed overnight at 30°C. Transcripts with a polyA+ tail were visualized using a 30-nt Cy3-oligo thymidyne probe at a concentration of 50 pmol/ml, and FISH was performed at 26°C. Control reactions were conducted without oligo probes. DNA was stained by 4,6-diamidino- 2-phenylindole (DAPI; Sigma-Aldrich).

**Measurement of chlorophyll content**

The total chlorophyll content was estimated according to Witham et al. . The samples consisting of 0,1g of tissue were extracted with 2 ml of 80% acetone. The supernatant was transferred to a volumetric flask after centrifugation at 5000 rpm for 5 min. The extraction was repeated until the residue was colorless. The absorbance of the extract was read at 645 and 663 nm against 80% acetone blank. The amount of total chlorophyll in the sample was calculated using the formula: Total chlorophyll = 20,2 × (A_645_) + 8,02 × (A_663_) × (V/(1000 × W)) where, V - final volume of the extract; W - weight of the leaves.

**Measurement of proline content**

Proline was measured following the methods of Abrahám et al. . The proline was extracted with 3% sulfosalicylic acid (5 µl/mg fresh weight). Next, 100 µl of the extract was mixed with 100 µl of 3% sulfosalicylic acid, 200 µl glacial acetic acid and 200 µl acidic ninhydrin. The mixture was incubated at 96°C for 60 min, terminated on ice and extracted with 1 ml toluene. The absorbance of the chromophore phase was measured at 520 nm against toluene as a reference.

**Detection of abscisic acid (ABA)**

The frozen leaves of *B. vulgaris* were homogenized in liquid nitrogen and mixed with 20 ml of 80% (v/v) methanol. Next the mixture was transferred to Erlenmeyer flasks and a small amount of anti-oxidant (BHT, butylhydroxytoluol) and 100 ng of deuterated ABA, [2H6]ABA, as internal standard, were added. The mixture was shaken overnight, then centrifuged and the supernatant was collected in evaporation flasks. The remaining pellet was resolved in 20 ml of 80% (v/v) methanol, shaken for 2 h and centrifuged again. Collected supernatants evaporated until all methanol was removed. Remaining aqueous fraction was acidified with HCl until pH 2 was reached. The extract was centrifuged and the supernatant was partitioned three times against ethyl acetate. The organic phase was collected in evaporation flasks and dried under vacuum. The dry residue was then resolved in 5 ml 1 M formic acid (FA) and loaded on a Discovery® DSC-18 SPE cartridge (Supelco Inc., USA). These cartridges were preconditioned with 4 ml methanol and allowed to equilibrate with 4 ml of 1 M FA. The columns were then subsequently washed with 4 ml 1 M FA, 4 ml of 20% methanol in 1 M FA and finally phytohormones were eluted with 4 ml of 80% methanol in water. The eluate was evaporated to dryness, resolved in 200 µl of 20% acetonitrile in water and applied to HPLC for further sample purification. HPLC was done with a SUPELCOSIL ABZ+ PLUS column (250 x 4.5 mm, 5 µm particle size; Supelco). The samples were chromatographed with a linear gradient of 20-80% acetonitrile in 0.1 M FA in 20 min, flow rate 1.0 ml/min. The fractions collected at 12 ± 0.5 min were evaporated to dryness, methylated overnight with diazomethane in diethylether. After this the diethylether was evaporated with N2 and the residue was dissolved in 30 μl of methanol. 1 µl of this sample was analysed by GC-MS (Auto-System XL coupled to a TurboMass, Perking-Elmer) using a MDN-5 column (30 mm x 0.25 mm, 0.25 µm phase thickness; Supelco). The GC temperature program was set at 60°C for 1 min, 60-250°C at 10°C/min, flow rate 1.5 ml/min, injection port was 280°C, electron potential 70 eV. The retention times of ABA and [^2^H6]ABA were 14.07 and 14.3 min, respectively. GC/MS was performed by monitoring m/z 190 for endogenous ABA and 194 for [^2^H6]ABA according to the method described by Vine et al. .

**Determination of physiological and morphological parameters**

Leaf gas exchange was determined using an LCpro+ portable photosynthesis system (ADC BioScientific, UK). Temperature, CO_2_ concentration, and irradiance in the leaf chamber during analysis were set to approximate ambient conditions. Relative chlorophyll content (chlorophyll content index, CCI) was measured with CCM-200 meter (Opti-Sciences, USA). Morphological characterization involved measurements of fresh mass of the above-ground part of plants, root length, leaf surface area and number. Root system of each plant was washed and its length was measured with a ruler. The leaf surface area was measured using a WinDIAS image analysis system (Delta-T Devices, UK).

**Relative water content (RWC)**

Relative water content was performed in the leaves of *B. vulgaris ssp. maritima* or *B. vulgaris* cv. *Huzar* subjected to salt stress treatment or salt shock treatments with salinity. The assay was performed as described by Ghoulam et al. . Five leaf discs of 10 mm diameter were excised from the interveinal areas of single leaves. Twenty discs were pooled and their FW is determined. Afterwards, the discs were floated in distilled water in Petri dishes for 4 h to regain turgidity, then blot-dried and re-weighed (TW). The samples were dried at 70*°*C for 24 h to determine the DW. RWC was defined as follows: RWC (%) = [(FW – DW)/(TW – DW)] *×* 100

**Leaf tissue processing for determination of mineral content of plants**

Preparing samples for P, K Mg, Na, Ca, Fe, Mn, Cu, Zn and B measurement involved initial rinsing with 0.01 M HCl, then washing in double-deionized water, drying at 60°C in a forced-draft oven (dryer with forced air circulation), and ground in a Wiley stainless steel mill. Finally the samples were microwave digested in HNO_3_, using closed Teflon vessels. Then the elements were determined by an inductively-coupled plasma spectrometer (ICP Model OPTIMA 2000DV, Perkin Elmer, USA). The plant material for nitrogen (N) analysis was dried, and its dry weight was determined by the drier oven method. Then the samples were mineralized in concentrated sulfuric acid in the presence of copper-potassium catalyst before the nitrogen content was determined using the Kijeldahl method. The measurement of N content by titration was performed after distillation of nitrogen in the form of NH^3^. Chlorides determined after  extraction with water by use the ion selective electrode model 9417 BN (Thermo Scientific, USA).

**The effects of salinity on mineral composition and properties of growth substrate solution**

Salt treatments slightly decreased the pH of the growth substrate solution, whereas its conductance greatly increased. The increase in the conductance of the medium apparently resulted from the increase in the Na and Cl (Fig. S3a,c). The concentrations of other elements, such as N, P, Mg and Ca were not affected by salt treatments. Potassium (K) content slightly, but significantly, decreased, following the treatment with strong salinity, but the moderate salinity did not affect the content of this element in the growth substrate solution (Fig. S3b,c).

**References**

1. Schmieder R, Edwards R. Quality control and preprocessing of metagenomic datasets. Bioinformatics 2011; 27:863-864.

2. Kim D, Pertea G, Trapnell C, Pimentel H, Kelley R, Salzberg SL. TopHat2: accurate alignment of transcriptomes in the presence of insertions, deletions and gene fusions. Genome Biology 2013; 14:R36.

3. Langmead B, Salzberg SL. Fast gapped-read alignment with Bowtie 2. Nature Methods 2012; 9:357-359.

4. Trapnell C, Roberts A, Goff L, Pertea G, Kim D, Kelley DR, Pimentel H, Salzberg SL, Rinn JL, Pachter L. Differential gene and transcript expression analysis of RNA-seq experiments with TopHat and Cufflinks. Nature Protocols 2012; 7:562-578.

5. Trapnell C, Hendrickson DG, Sauvageau M, Goff L, Rinn JL, Pachter L. Differential analysis of gene regulation at transcript resolution with RNA-seq. Nature Biotechnology 2013; 31:46-53.

6. Alexa A, Rahnefuhrer J. topGO: Enrichment analysis for Gene Ontology. 2010.

7. Alexa A, Rahnenfuhrer J, Lengauer T. Improved scoring of functional groups from gene expression data by decorrelating GO graph structure. Bioinformatics 2006; 22:1600-1607.

8. Skorupa-Kłaput M, Szczepanek J, Kurnik K, Tretyn A, Tyburski J. The expression patterns of plasma membrane aquaporins in leaves of sugar beet and its halophyte relative, *Beta vulgaris ssp. maritima*, in response to salt stress. Biologia 2015; 70:467–477.

9. Pfaffl MW, Horgan GW, Dempfle L. Relative expression software tool (REST) for group-wise comparison and statistical analysis of relative expression results in real-time PCR. Nucleic Acids Research 2002; 30:e36.

10. Tirichine L, Andrey P, Biot E, Maurin Y, Gaudin V. 3D fluorescent in situ hybridization using *Arabidopsis* leaf cryosections and isolated nuclei. Plant Methods 2009; 5:11.

11. Witham F, Blaydesand D, Devlin R: Experiments in plant physiology. New York: Van Nostrend Reinhold Company; 1971.

12. Abraham E, Hourton-Cabassa C, Erdei L, Szabados L. Methods for determination of proline in plants. Methods in Molecular Biology 2010; 639:317-331.

13. Vine JH, Niton D, Plummer JA, Baleriola-Lucas C, Mullins MG. Simultaneous quantitation of indole-3-acetic acid and abscisic acid in small samples of plant tissue by gas chromatography/mass spectrometry/selected ion monitoring. Plant Physiology 1987; 85:419-422.

14. Ghoulam C, Foursy A, Fares K. Effects of salt stress on growth, inorganic ions and proline accumulation in relation to osmotic adjustment in five *s*ugar beet cultivars. Environmental and Experimental Botany 2002; 47:39-50.
